# Inside-out Ca^2+^ signalling prompted by STIM1 conformational switch

**DOI:** 10.1038/ncomms8826

**Published:** 2015-07-17

**Authors:** Guolin Ma, Ming Wei, Lian He, Chongxu Liu, Bo Wu, Shenyuan L. Zhang, Ji Jing, Xiaowen Liang, Alessandro Senes, Peng Tan, Siwei Li, Aomin Sun, Yunchen Bi, Ling Zhong, Hongjiang Si, Yuequan Shen, Minyong Li, Mi-Sun Lee, Weibin Zhou, Junfeng Wang, Youjun Wang, Yubin Zhou

**Affiliations:** 1 Institute of Biosciences and Technology, Texas A&M University Health Science Center, Houston, Texas 77030, USA; 2 Beijing Key Laboratory of Gene Resource and Molecular Development, College of Life Sciences, Beijing Normal University, Beijing 100875, China; 3 High Magnetic Field Laboratory, Hefei Institutes of Physical Science, Chinese Academy of Sciences, Hefei 230031, China; 4 University of Science and Technology of China, Hefei, Anhui 230036, China; 5 Department of Medical Physiology, College of Medicine, Texas A&M University Health Science Center, Temple, Texas 76504, USA; 6 Department of Biochemistry, University of Wisconsin Madison, Madison, Wisconsin 53706, USA; 7 College of Life Sciences, Nankai University, Tianjin 300071, China; 8 Key Laboratory of Chemical Biology, School of Pharmacy, Shandong University, Jinan, Shandong 250012, China; 9 Department of Pediatrics and Communicable Diseases, University of Michigan Medical School, Ann Arbor, Michigan 48109, USA

## Abstract

Store-operated Ca^2+^ entry mediated by STIM1 and ORAI1 constitutes one of the major Ca^2+^ entry routes in mammalian cells. The molecular choreography of STIM1–ORAI1 coupling is initiated by endoplasmic reticulum (ER) Ca^2+^ store depletion with subsequent oligomerization of the STIM1 ER-luminal domain, followed by its redistribution towards the plasma membrane to gate ORAI1 channels. The mechanistic underpinnings of this inside-out Ca^2+^ signalling were largely undefined. By taking advantage of a unique gain-of-function mutation within the STIM1 transmembrane domain (STIM1-TM), here we show that local rearrangement, rather than alteration in the oligomeric state of STIM1-TM, prompts conformational changes in the cytosolic juxtamembrane coiled-coil region. Importantly, we further identify critical residues within the cytoplasmic domain of STIM1 (STIM1-CT) that entail autoinhibition. On the basis of these findings, we propose a model in which STIM1-TM reorganization switches STIM1-CT into an extended conformation, thereby projecting the ORAI-activating domain to gate ORAI1 channels.

## Abstract (web summary)

Depletion of calcium from intracellular stores induces interaction between the endoplasmic reticulum STIM1 protein and the plasma membrane ORAI1 channel that facilitates cellular calcium entry. Here Ma *et al*. characterize a STIM1 gain-of-function mutant and propose a conformational switch that controls ORAI1 gating.

## Graphical abstract

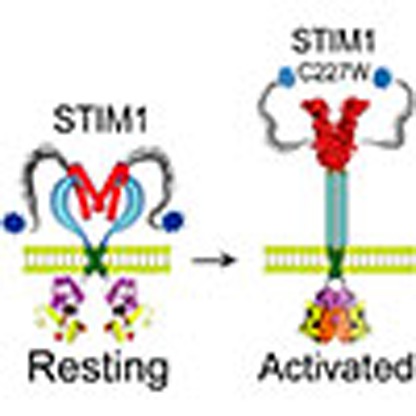

T-cell receptor engagement by antigens is an instrumental step in initiating the host adaptive immune response against invading pathogens^1^. It triggers a cascade of signalling events to elicit the influx of extracellular Ca^2+^ through the Ca^2+^ release-activated Ca^2+^ (CRAC) channel, a classical example of store-operated calcium entry (SOCE) that is mediated by the stromal interaction molecule 1 (STIM1) and ORAI1 (ref. 2). Aberrant STIM–ORAI signalling is intimately involved in the pathogenesis of immunodeficiency, cardiac hypertrophy and cancer metastasis^2^,^3^,^4^,^5^,^6^. The dynamic STIM1–ORAI1 coupling represents a unique inside-out signalling event that occurs at endoplasmic reticulum (ER)–plasma membrane (PM) junctions. We and others have previously shown that the four-pass transmembrane protein ORAI forms the pore subunit of the CRAC channel^7^,^8^ and is directly gated by an ER-resident Ca^2+^ sensor protein STIM1 through physical interactions^6^,^9^,^10^,^11^,^12^,^13^,^14^,^15^. The major activation steps for SOCE have thus far been understood at a descriptive level^2^,^5^,^15^. Following store depletion, the dissociation of Ca^2+^ from the luminal EF-hand motif induces dimerization or oligomerization of the STIM1 luminal domain. It is generally assumed that this switches STIM1 into an extended conformation, with subsequent migration of STIM1 towards regions of ER–PM appositions (termed ‘puncta’) to open ORAI1 Ca^2+^ channels through physical contacts.

Although considerable insight into the STIM–ORAI pathway has been gained from cellular studies (reviewed in refs 2, 3, 4, 5, 15), the mechanistic underpinnings of this inside-out Ca^2+^ signalling, particularly the crucial questions of how ER-luminal signals are transmitted towards the STIM1 cytoplasmic domain (STIM1-CT) and how the STIM1 juxtamembrane coiled-coil region (CC1) interplays with the ORAI-activating domain (CAD/SOAR)^9^,^10^, have not been addressed. This is largely because of the technical difficulty in capturing and stabilizing resting and activated STIM1 in a membrane-like environment *in vitro*, and the lack of robust assays to visualize the CC1–SOAR interaction at real time under physiological conditions. Here we have adopted a ‘divide-to-conquer’ strategy with a focus on the interplays among the single transmembrane domain of STIM1, the juxtamembrane cytosolic coiled-coil (CC1) region and the minimal ORAI1-activating domain CAD/SOAR^9^,^10^. We overcome the first hurdle by taking advantage of a previously unrecognized ‘gain-of-function’ mutation within the single transmembrane domain of STIM1 (STIM1-TM) and reconstitution of STIM1-TM into bicelles/nanodiscs that are compatible with a variety of biophysical assays. We show, first, that the mutant C227W represents STIM1 in one of its activated states, which enables us to probe the conformational changes within the transmembrane domain both *in vitro* and *in vivo*. With chemical cross-linking and high-resolution nuclear magnetic resonance (NMR), we pinpoint critical residues involved in the reorganization of STIM1-TM during its activation. Next, we show that local rearrangement, rather than alterations in the oligomeric state of STIM1-TM, is sufficient to prompt conformational changes in the juxtamembrane CC1 region. As a consequence, CC1 adopts a more extended conformation and assumes more helical contents presumably by forming a stabilized coiled coil. To tackle the second obstacle, we have devised a novel fluorescence resonance energy transfer (FRET)-based assay to monitor the dynamic CC1–SOAR interaction under physiological conditions. The two-component FRET system affords a real-time visualization of CC1–SOAR interaction, as well as the ‘tug-of-war’ between CC1 and ORAI1 in living cells. This robust assay also opens a new avenue to fine-map the CC1–SOAR contact sites, which keeps SOAR quiescent at rest. Overall, our study provides important mechanistic insights into a conformational switch model of SOCE.

## Results

### Identification of new gain-of-function mutations in STIM1-TM

**Figure 1:**
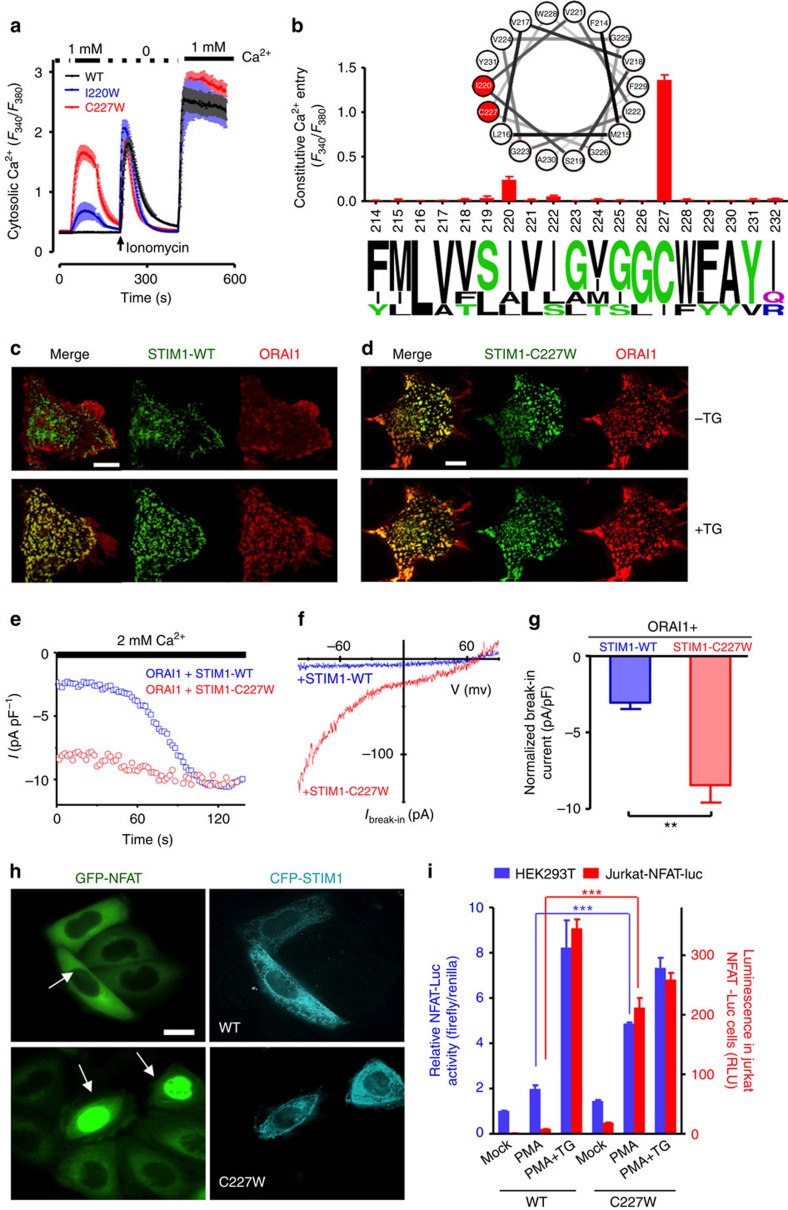
Identification of novel gain-of-function mutations within STIM1-TM. (**a**) Ca^2+^ influx in HEK ORAI1-CFP stable cells expressing WT and mutant STIM1-TM constructs (*n*=30–60) monitored by Fura-2 fluorescence ratio. Store depletion was induced by 2.5 μM ionomycin. Shown are representative traces from three independent experiments. The solid bar above the curves indicates 1 mM Ca^2+^ in the external medium. (**b**) Effects of tryptophan substitution of STIM1-TM residues on constitutive Ca^2+^ entry in HEK ORAI1-CFP stable cells. The level of constitutive Ca^2+^ entry (*n*=3) was quantified as the difference of mean Fura-2 fluorescence ratio between the peak value in the presence of 1 mM Ca^2+^ and the basal value without externally added Ca^2+^. The size of letters beneath the *x* axis represents the probability of conserved residues at each position across species including humans, mice, worms and flies. Inset, helical wheel projection of an ideal STIM-TM helix. (**c**,**d**) Confocal images of footprint of HeLa cells co-transfected with mCherry-ORAI1 and WT (**c**) or C227W-EGFP-STIM1 (**d**) constructs. Thapsigargin (TG), 1 μM, was added to trigger store depletion. Scale bar, 5 μm. (**e**–**g**) Constitutive activation of CRAC channels by overexpression of STIM1-C227W. (**e**) Two representative time courses of normalized inward currents (pA/pF), measured at −100 mV in HEK293 cells co-expressing ORAI1+WT STIM1 (blue) or ORAI1+STIM1-C227W (red), respectively. Strongly pre-activated CRAC currents were observed in the cells transfected with ORAI1+STIM1-C227W. (**f**) Corresponding leak-subtracted *I*–*V* curves taken from the break-in time points (*t*=0 s). (**g**) Normalized break-in currents averaged from cells transfected with ORAI1+WT STIM1 (*n*=5) or ORAI1+STIM1-C227W (*n*=6). (**h**) Confocal images of GFP-NFAT1 stable HeLa cells (left panels) transiently transfected with WT (right panel, top) or C227W (right panel, bottom) CFP-STIM1 constructs without store depletion. Note that cells expressing CFP-STIM1-C227W (arrow), but not WT CFP-STIM1, led to constitutive GFP-NFAT nuclear translocation. Scale bar, 5 μm. (**i**) Quantification of NFAT-dependent luciferase activity in HEK293T cells or Jurkat (NFAT-Luc) cells transfected with WT or C227W STIM1 constructs. PMA, phorbol 12-myristate 13-acetate. **P<0.01, ***P<0.001, Student’s *T*-test. Error bars in all panels are defined as s.e.m.

Ca^2+^ influx through the ORAI1 channel is often conveniently triggered through passive store depletion with thapsigargin (TG) or the Ca^2+^ ionophore ionomycin in model cellular systems such as HeLa or HEK293 cells^16^. However, mutations causing loss of Ca^2+^-sensing capability (for example, D76A)^17^ or perturbing the structural integrity of the cytosolic domain (for example, L251S and R304W)^18^,^19^ can bypass this manipulation and cause constitutive STIM1 puncta formation and Ca^2+^ influx through CRAC channels. These types of ‘gain-of-function’ mutations are thought to largely represent activated conformations of STIM1, but the existence of such mutations in the STIM1 transmembrane domain has yet to be explored. Given this unique structural and functional feature, we set out to screen constitutively activating mutations throughout STIM1-TM by taking a tryptophan-scanning approach^20^,^21^, with the hope of introducing steric hindrance at the contact interface to affect STIM1-TM helix packing and thus cause functional abnormality. Our efforts led to the discovery of at least two previously unrecognized ‘gain-of-function’ mutations (that is, I220W and C227W), both of which could be mapped to the same side of a projected STIM1-TM helical wheel (Fig. 1a,b). These mutations did not seem to alter Ca^2+^ release from ER stores or the second phase of ionomycin-triggered Ca^2+^ influx in the typical ‘Ca^2+^ add-back’ experiment^22^ (Fig. 1a; Supplementary Fig. 1a,b). The gain-of-function phenotype, albeit at varying degrees of constitutive activation, could also be recapitulated by substituting C227 with several other amino acids, including asparagine, glutamine, histidine, glutamate, tyrosine and aspartate (Supplementary Fig. 1c), or in HEK293 cells without overexpression of ORAI1 (Supplementary Fig. 1d). Since C227W exhibited the highest potency to elicit constitutive Ca^2+^ influx, we focused our following experiments on this unique mutation.

To unequivocally confirm the ‘gain-of-function’ phenotype of C227W, we examined the behaviour of this mutant in the context of several critical steps of the Ca^2+^/nuclear factor of activated T-cell (NFAT) signalling pathway^2^. Compared with wild type (WT), STIM1-C227W formed puncta at resting condition (Fig. 1c,d) and elicited constitutive Ca^2+^ influx through CRAC channels as reflected by spontaneous development of CRAC currents (*I*_CRAC_) without store depletion (Fig. 1e–g). Notably, STIM1-C227W expression prompted NFAT translocation from the cytosol to the nucleus (Fig. 1h) and further induced NFAT-dependent expression of luciferase in both HEK293T cells and Jurkat T cells without store depletion (Fig. 1i). Thus, C227W represents an activated state of STIM1 and is well suited to serve as a unique molecular tool to dissect the activation steps of SOCE by circumventing direct disruption of structural integrity of the luminal domain or the cytoplasmic domain.

One notable feature of STIM1-TM is the presence of three glycine residues with small side chains. Glycine residues within a transmembrane domain often participate in close helix–helix packing or the introduction of kinks^23^. We reason that small-to-large substitution of multiple glycines in STIM1-TM would impact its structural rearrangement and thus compromise its normal activity. Indeed, replacement of three glycines or G223/G225 by tryptophans significantly reduced SOCE (Supplementary Fig. 1e). Together single or multiple mutations within STIM1-TM could lead to either gain-of-function or hypomorphic phenotypes, prompting us to further dissect how structural changes in STIM1-TM facilitate the signal transduction from the ER lumen to the cytoplasm.

### STIM1-C227W prompts structural reorganization of STIM1-TM

**Figure 2:**
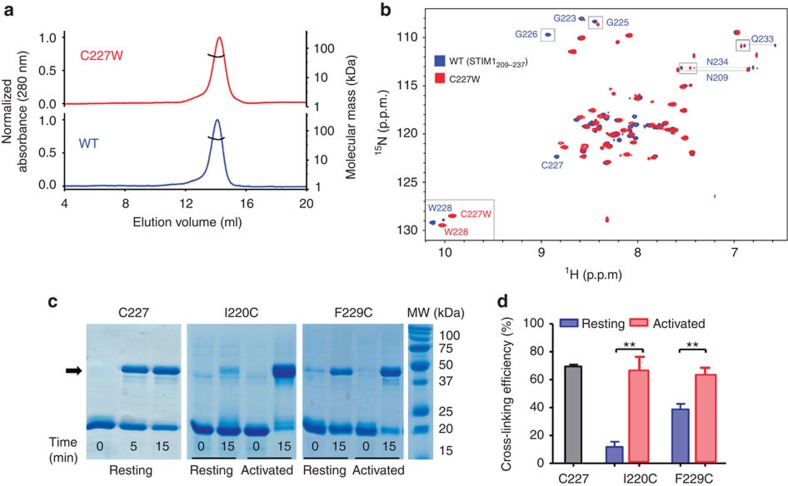
Structural changes of STIM1-TM domain upon activation. (**a**) SEC-MALLS profiles of WT (blue, resting) or C227W (red, activated state) TM-CC1 (STIM1_209–310_) proteins. The recombinant protein migrated as a single symmetric peak with the plotted molecular size referring to the axis at the right. Both proteins (MW_theoretical_: 21 kDa; MW_experimental_: 49∼50 kDa) were N-terminally fused with a monomeric GB1 tag to aid purification. (**b**) Superimposed ^15^N–^1^H HSQC spectra of WT (blue) and C227W (red) STIM1-TM (residue 209–237) reconstituted into bicelles. Grey box, representative residues undergoing chemical shift changes between the resting (WT) and activated (C227W) conformations. (**c**,**d**) CuP-catalysed cross-linking of selected TM-CC1 monocysteine variants. (**c**) Disulfide cross-linking is reflected as a dimer band (arrow) on the non-reducing SDS–PAGE. (**d**) Bar graph shows the cross-linking efficiency of STIM1-TM monocysteine variants (*n*=3). **P<0.01, Student’s *T*-test. Error bars in panels are defined as s.e.m.

To examine how the activating mutation affects the oligomeric state of STIM1-TM, we compared the molecular mass between recombinant WT and C227W in the context of TM-CC1 (residues 209–310) by size-exclusion chromatography coupled with multi-angle laser light scattering. Both WT and C227W eluted in sizes comparable to dimer under our conditions *in vitro* (Fig. 2a; Supplementary Fig. 2a). Clearly, this result provides strong evidence arguing against the possibility of a monomer-to-oligomer transition within the transmembrane domain when STIM1 switches into an activated status. Nonetheless, since the recombinant proteins only contain the TM-CC1 region, it does not exclude the involvement of other regions (for example, EF-SAM or CAD/SOAR) in driving the formation of higher-order oligomerization during STIM1 activation. Next, we carried out high-resolution NMR studies on bicelle-reconstituted STIM1-TM to pinpoint residues that undergo substantial structural changes during STIM1 activation, which will be very likely reflected in the chemical shift perturbation. We found that residues including G225, G226, W228, Q233 and N234 displayed significant resonance changes when STIM1 adopted an activated conformation (Fig. 2b).

To further reveal potential conformational changes within STIM1-TM, we decided to use the substituted cysteine-scanning approach coupled with copper–phenanthroline (CuP) cross-linking across the predicted STIM1 transmembrane domain, an assay that we previously developed to probe the ORAI1 pore architecture^13^. This method proves to be a convenient and reliable way to dissect inter-subunit contacts and probe conformational changes in transmembrane helices. The changes in cross-linking efficiency could arise from distance change due to helical swivelling or relative movement towards one another. We applied this chemical cross-linking approach to compare the conformations of STIM1-TM at resting or activated states. Since unambiguous analyses of cross-linking patterns require a cysteine-less background with subsequent introduction of monocysteine at each position along STIM1-TM, we used C227S to mimic the WT-like resting state, given their similarity in side-chain size and polarity, while C227W represents an activated status of STIM1-TM. Prior to cross-linking, the monocysteine mutants predominantly migrated as a single band corresponding to the size of the monomer on non-reducing SDS–polyacrylamide gel electrophoresis (PAGE) (Fig. 2c,d). Upon addition of CuP, recombinant STIM1-TM proteins displayed a second band, indicating cross-linking of disulfide bonds. The cross-linking data suggest that STIM1-TM possesses two distinct segments with varying conformational flexibility. In the C-terminal region, the cross-linking efficiency is generally high at all positions, possibly due to flexibility induced by the multiple glycine residues and likely enhanced in a detergent environment. The cross-linking pattern displays a periodicity that is consistent with the periodicity of an ideal α-helix. A230C, C227, G226C and I222C exhibited over 70% disulfide bond formation (Supplementary Fig. 3a), suggesting that these positions are probably located at the dimer interface. By contrast, the N-terminal segment of STIM1-TM (residues 214–220) displayed markedly lower cross-linking efficiency (Supplementary Fig. 3a). This may indicate that the helices of the STIM1-TM dimer interact prevalently through the C-terminal positions, and that a wide crossing angle of the two helices might splay the N-terminal positions apart. This conformation is consistent with a computational model of the STIM1-TM dimer obtained with the program CATM^19^, which predicts that STIM1-TM is compatible with a GAS_right_ dimer, a frequent structural motif in membrane proteins mediated by GxxxG-like sequence patterns^24^ (in the case of STIM1, the dimer is predicted to be mediated by S_219_xxxG_223_xxxC_227_, Supplementary Fig. 3b). Compared with C227S variants, at least two monocysteine variants (for example, I220C and F229C) in the cysteine-less background of STIM1-TM-C227W displayed significant changes in the cross-linking efficiency (Fig. 2c,d). Thus, we conclude that STIM1-TM undergoes structural rearrangements when adopting an activated conformation.

**Figure 3:**
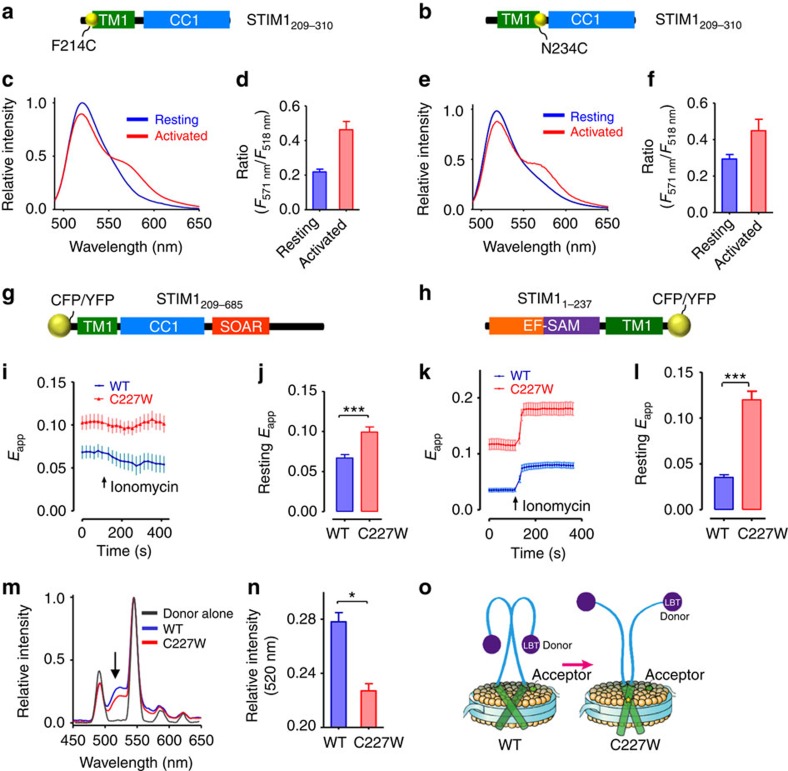
Structural changes of STIM1-TM-CC1 upon activation. (**a**–**f**) Positions for donor (FITC)–acceptor (TMR) labelling on F214C (**a**) and N234C (**b**) are indicated. Fluorescence spectra (*λ*_exc_=475 nm, **c**,**e**) and quantification (**d**,**f**) of FRET signals for labelled monocysteine variants (**c**,**d**: for F214C; **e**,**f**: for N234C) in the context of C227S (blue) or C227W (red) TM-CC1. FRET ratio is presented as mean±range of two independent experiments. (**g**–**l**) FRET signals monitored before and after ionomycin-induced store depletion in HEK293 cells co-expressing (**i**,**j**) CFP/YFP-STIM1_209–685_ or (**k**,**l**) STIM1_1–237_-CFP/YFP. The resting FRET signals are plotted as a bar graph. Schematic of donor (CFP)–acceptor (YFP) labelling is shown in **g** and **h**. (*n*=3). (**m**–**o**) STIM1-TM rearrangement prompts extension of the juxtamembrane CC1 region. LRET measurements were made on TM-CC1 with its C terminus fused to a genetically encoded LBT tag. Tb^3+^-bound LBT serves as the donor to form LRET pair with a FITC-labelled phospholipid analogue incorporated into the nanodisc. (**m**) Gated luminescence spectra of labelled WT (blue) or C227W (red) TM-CC1 nanodiscs (*λ*_exc_=280 nm). TM-CC1-LBT was also assembled into non-fluorescent nanodiscs to acquire the donor alone spectrum (grey). Arrow indicates FITC acceptor emission arising from energy transfer. (**n**) Quantification of LRET signals at the acceptor emission peak (520 nm). (**o**) Cartoon interpreting the results. LBT, lanthanide-binding tag. *P<0.05, ***P<0.001 Student’s *T*-test. Error bars denote s.e.m.

We sought further confirmation that STIM1-TM undergoes conformational change with another independent assay *in vitro*. We introduced donor and acceptor fluorophores at either end of the dimeric STIM-TM through specific labelling at a single cysteine (that is, position F214C or N234C). We then compared the FRET signals at these two positions between the resting and activated states. After switching to an activated conformation as exemplified by C227W, we observed an increase in the FRET signal (Fig. 3a–f), suggesting a closer spatial proximity of the donor and acceptor at both the N- and C-terminal end of STIM1-TM. This was further confirmed through parallel cellular FRET assays by fusing cyan fluorescent protein (CFP) or yellow fluorescent protein (YFP) to each end of the STIM1 transmembrane domain (Fig. 3g–l). The activating mutant C227W displayed significantly higher FRET signals than WT, thus reinforcing the conclusion that both ends of STIM1-TM approach closer in space or undergo helical rotations in response to STIM1 activation.

### STIM1-TM reorganization causes conformational changes in CC1

The above findings led us to explore the conformational switch hypothesis that STIM1-TM reorganization might prompt further conformational changes in the cytosolic juxtamembrane region^5^. We therefore extended our studies from STIM1-TM to encompass the juxtamembrane coiled-coil region (TM-CC1). Compared with WT TM-CC1, the activating mutant C227W showed ∼12% increase in the overall helical content (Supplementary Fig. 2b,c) in the secondary structure. The helical structure change could be attributed to the CC1 region, since the secondary structures of STIM1-TM in resting or activated states remain unaltered (Supplementary Fig. 2d). These results are in agreement with our previous finding that bringing the N termini of CC1 together through forced cross-linking led to increased helicity in CC1, presumably due to the formation of coiled coil in the initial region of CC1 (ref. 6).

To more faithfully mimic its behaviour in a membrane-like environment, recombinant TM-CC1 protein was incorporated into nanodiscs, in which islands of lipid bilayer are surrounded by a belt of engineered apolipoprotein A1 protein (Supplementary Fig. 4a)^25^. We decided to use the Tb^3+^-acceptor luminescence resonance energy transfer (LRET), a sensitive assay that we have successfully applied to estimate the head-to-end distance of STIM1-CT^6^, to reveal conformational changes that might be reflected in the relative distance change between the lipid bilayer and the C terminus of CC1. TM-CC1 nanodisc with donor alone exhibited the characteristic emission spectrum of excited Tb^3+^ (Fig. 3m–o). TM-CC1 nanodisc with both donor and acceptor displayed an additional emission peak with the maximum at ∼520 nm, indicating the energy transfer from the donor Tb^3+^ to the acceptor FITC residing on the nanodisc (Fig. 3m–o). By contrast, the gated spectrum of TM-CC1-C227W nanodisc showed a relatively lower emission peak centred around 520 nm. To exclude potential bias introduced during LRET measurements, we subsequently swapped the position of the donor–acceptor pair (Supplementary Fig. 4b). Again, we noticed a reduction of FRET signals in TM-CC1-C227W nanodisc with diminished acceptor emission and concomitant increase in donor emission. Collectively, results from these two complementary assays suggest that STIM1-CC1 adopts a more extended conformation possibly by gaining more helical content and reorienting its C terminus away from the ER membrane following apposition of the N termini of STIM1-CT induced by STIM-TM rearrangement.

### L258-L261 in CC1 is critical for its docking with SOAR

**Figure 4:**
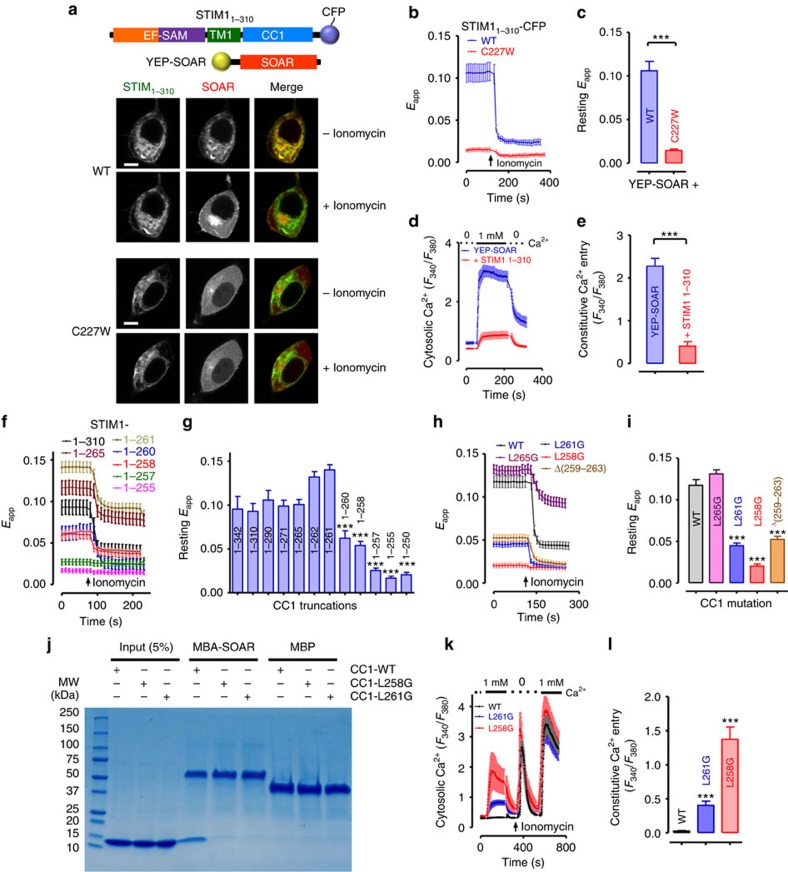
Visualization of CC1–SOAR interplays and mapping of SOAR-docking sites within CC1. (**a**) Confocal images of HEK293 cells co-expressing YFP-SOAR and WT (upper panel) or C227W (lower panel) STIM1_1–310_-CFP constructs. Ionomycin (2.5 μM) was added to trigger store depletion. In merged images, CFP and YFP signals are shown in red and green pseudocolour, respectively, to aid better visualization. Scale bar, 5 μm. Schematic of donor (CFP)–acceptor (YFP) labelling is shown on the top. (**b**,**c**) Real-time FRET signals monitored before and after ionomycin-induced store depletion in HEK293 cells co-expressing STIM1_1–310_-CFP and YFP-SOAR (**b**). (**c**) The resting FRET signals are plotted as a bar graph. (**d**,**e**) Overexpression of STIM1_1–310_-CFP suppressed constitutive Ca^2+^ influx induced by YFP-SOAR in HEK ORAI1 stable cells (**d**). The solid bar above the curves indicates 1 mM Ca^2+^ in the external medium. (**e**) The mean constitutive Ca^2+^ flux is plotted as a bar graph. (**f**–**i**) Mapping SOAR-docking sites on CC1. Serial truncation of the C terminus (**f**,**g**) or introduction of selected mutations (**h**,**i**) in STIM1_1–310_ enables us to delineate a minimal docking region for the isolated SOAR/CAD domain. FRET signals monitored before and after ionomycin-induced store depletion in HEK293 cells co-expressing YFP-SOAR with truncated variants (**f**), or with the indicated mutants (**h**) derived from STIM1_1–310_-CFP. (**g**,**i**) The resting FRET signals are plotted as a bar graph. Note that similar trends can be recapitulated in cells co-expressing mCherry-CAD/SOAR and STIM1_1–310_-CFP variants (Supplementary Figs 5 and 6). (**j**) Binding of recombinant STIM1-CC1 (233–310), CC1-L258G and CC1-L261G to recombinant MBP-SOAR proteins immobilized on amylose resin. Introduction of mutations L258G or L261G in CC1 abolished CC1–SOAR interaction *in vitro*. Maltose-binding protein (MBP) was used as a negative control. (**k**,**l**) Ca^2+^ influx in HEK ORAI1-CFP stable cells expressing WT and mutant full-length STIM1 constructs monitored by Fura-2 fluorescence ratio (**k**). The solid bar above the curves indicates 1 mM Ca^2+^ in the external medium. (**l**) The mean constitutive Ca^2+^ flux is plotted on the right as a bar graph. ***P<0.001, Student’s *T*-test. All error bars denote s.e.m. for at least three independent experiments with 15–25 cells.

The next question we asked is how extended CC1 affects the activity of the ORAI-activating SOAR/CAD domain. There is a tacit belief that SOAR/CAD is kept quiescent at rest but will be activated to engage ORAI1 channels following store depletion^2^,^3^,^4^,^5^,^6^. Although we had previously demonstrated the physical interaction between recombinant CC1 and SOAR^6^ and determined the CC1–SOAR-binding affinity *in vitro* (*K*_D_=9.4 μM, Supplementary Fig. 2g), the underlying structural determinants remain elusive. To solve this puzzle under physiologically relevant conditions, we developed a FRET assay by fusing donor or acceptor fluorophores to two autonomous components of STIM1 (Fig. 4a). This two-component system affords the first real-time visualization of CC1–SOAR/CAD interaction, as well as dissociation induced by store depletion and the ‘tug-of-war’ between CC1 and endogenous ORAI1 (Fig. 4; Supplementary Fig. 5; Supplementary Movies 1–4). Under resting condition, isolated SOAR/CAD was tightly tethered against the ER membrane in cells expressing STIM1_1–310_ (Fig. 4a) or STIM1_1–342_ (Supplementary Fig. 5c), and exhibited an ER-like tubular distribution pattern. Moreover, overexpression of STIM1_1–310_ led to a significant suppression of SOAR-induced constitutive Ca^2+^ influx (Fig. 4d,e). Following store depletion, the majority of SOAR/CAD was dispersed into the cytosol, thus resulting in a remarkable drop in FRET signals and enabling its subsequent interaction with ORAI1 (Fig. 4a–c; Supplementary Fig. 5c; Supplementary Movies 1 and 2). By contrast, SOAR/CAD displayed an even cytosolic distribution regardless of the ER Ca^2+^ filing status in cells expressing the activating mutant STIM1_1–310_-C227W (Fig. 4a–c; Supplementary Movie 3).

The substantial FRET signal change provides an excellent opportunity for us to unambiguously map out the minimal region within CC1 essential for SOAR docking. Through a series of deletion and mutagenesis studies, we narrowed down the possible SOAR-interacting site to residues 255–265 with complementary FRET (Fig. 4f–i), colocalization assays (Supplementary Figs 5 and 6), *in vitro* pull-down (Fig. 4j and binding assays (Supplementary Fig. 2g)). In particular, residues 258–261 are crucial for the CC1–SOAR interaction, since CC1–SOAR interaction was severely compromised with the deletion of residues 259–263, or with the introduction of mutations L258G or L261G (Fig. 4h,i; Supplementary Fig. 6a,b). Substitution of leucine with alanine at position 258, which presumably would retain the helical propensity, also led to a significant decrease in the FRET signals at rest (Supplementary Fig. 7a). These mutant constructs caused spontaneous puncta formation (Supplementary Fig. 6c) and constitutive Ca^2+^ influx in HEK293 cells (Fig. 4k,l).

### SOAR but not D-SOAR forms heteromers with STIM_1–442_

**Figure 5:**
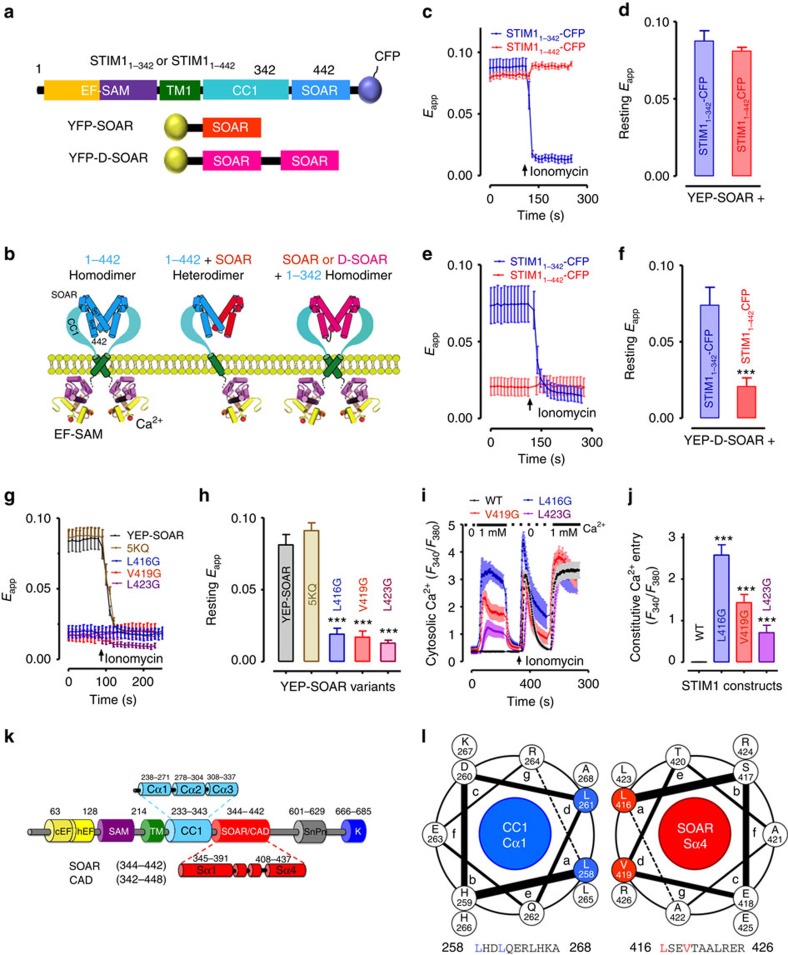
Molecular determinants within SOAR required for docking to CC1. (**a**,**b**) Schematic of donor (CFP)–acceptor (YFP) labelling (**a**) and cartoon interpreting the results (**b**). Isolated SOAR, but not D-SOAR, readily forms a heterodimer with STIM1_1–442_. Nonetheless, both isolated SOAR and D-SOAR could dock to STIM1_1–342_. The relative positions of TM, CC1 and SOAR remain to be determined. (**c**–**f**) FRET signals monitored before and after ionomycin-induced store depletion in HEK293 cells co-expressing YFP-SOAR (**c**) or YFP-D-SOAR (**e**) with STIM1_1–342_-CFP (blue) or STIM1_1–442_-CFP (red). The resting FRET signals are plotted as a bar graph (**d**,**f**). (**g**,**h**) FRET signals monitored before and after ionomycin-induced store depletion in HEK293 cells co-expressing STIM1_1–310_-CFP and YFP-SOAR or indicated mutant constructs. (**i**,**j**) Ca^2+^ influx in HEK ORAI1-CFP stable cells expressing WT and mutant full-length STIM1 constructs. The solid bar above the curves indicates 1 mM Ca^2+^ in the external medium (**i**). The mean of constitutive Ca^2+^ flux is plotted as a bar graph (**j**). (**k**,**l**) Schematic of the proposed coiled-coil interplays between CC1-Cα1 and the SOAR-Sα4 helices. (**k**) Domain architecture of STIM1. cEF, canonical EF-hand; hEF, hidden EF-hand, TM, transmembrane domain. Putative coiled-coil 1 (CC1); the minimal ORAI-activating region (SOAR/CAD); Pro/Ser-rich region (SnPn); and the polybasic C-tail (K). (**l**) Proposed coiled-coil interface formed by the CC1 α-1 helix (Cα1) and the SOAR α-4 helix (Sα4). ***P<0.001, Student’s *T*-test. All error bars denote s.e.m. for at least three independent experiments.

By slightly modifying the donor/acceptor pairs in the FRET assay, we were also able to further dissect the molecular determinants within SOAR that govern its docking to CC1 (Fig. 5). We first characterized the oligomeric status of 1–342 and 1–442 *in vitro*. Elution profiles from size-exclusion chromatography (Supplementary Fig. 8a,b) and gel electrophoresis on native PAGE (Supplementary Fig. 8c) suggested that both proteins predominately existed as a dimer, regardless of the presence or absence of Ca^2+^. Our attempts to capture the 1–342/SOAR or 1–442/SOAR complexes *in vitro* were not successful, because the dimeric SOAR tends to fall apart and only stays as a monomer in the presence of detergents (dashed line, Supplementary Fig. 2f), the latter of which is required to keep 1–342 or 1–442 soluble and stable in aqueous solution. We then compared the docking behaviour of isolated CAD/SOAR domain and D-SOAR (two copies of SOAR covalently linked in tandem) toward 1–342 or 1–442 in the cellular FRET assay (Fig. 5a–f). We found that the isolated CAD/SOAR domain docked tightly to STIM1_1–442_ regardless of the fluctuation of ER Ca^2+^ (Fig. 5c,d; Supplementary Fig. 9), a phenomenon that can be attributed to the formation of stable SOAR heteromers. To the contrary, D-SOAR failed to associate with STIM1_1–442_, a construct that contains an intact SOAR domain by itself, but it still retained the ability to interact with STIM1_1–342_ (Fig. 5e,f, blue line) or the further truncated variant STIM1_1–310_ at resting conditions (Supplementary Fig. 10). This finding strongly implicates that SOAR may function in a dimeric form since only isolated SOAR domain, rather than the concatemeric D-SOAR, is capable of forming a heteromeric complex with the CAD/SOAR fragment in the context of STIM1_1–442_ (Fig. 5b). This argument is further corroborated by our finding that truncation of a region (344–349) involved in SOAR dimerization or introduction of double mutations L347A/Q348A in this region^10^,^26^ led to failure of YFP-SOAR docking towards CC1 in the contexts of STIM1_1–342_ or STIM1_1–442_ (Supplementary Fig. 9b,c).

### Mapping residues critical for the CC1–SOAR association

In an attempt to map the critical residues in SOAR for its association with CC1, we focused on the two long helices (Sα1 and Sα4, Fig. 5k,l) in SOAR that have high potential to form coiled coils. Mutations in the Sα1 helix (^382^KIKKKR^387^>QIQQQQ, termed 5KQ) failed to abolish CC1–SOAR interaction (Fig. 5g,h). Notably, 5KQ mutations are known to abrogate the coupling of SOAR to ORAI1 channels^27^,^28^. Thus, the CC1 contact sites within SOAR are likely distinct from its binding sites with ORAI1. Nonetheless, residues L416, V419 and possibly L423 within the Sα4 helix seemed to be crucial for CC1–SOAR interaction, since disruption of coiled-coil formation through substitution with glycine (Fig. 5g,h) or alanine (Supplementary Fig. 7a) at these positions caused dissociation of SOAR/CAD from CC1, spontaneous STIM1 puncta formation (Supplementary Fig. 9e) and constitutive Ca^2+^ influx (Fig. 5i,j). All these mutations did not seem to perturb the dimerization of SOAR by judging from their capacity to interact with STIM1_1–442_ through heterodimerization (Supplementary Fig. 9d) and from the *in vitro* gel filtration elution profiles (Supplementary Fig. 2f). To conclude, results from our FRET and colocalization assays unveiled an additional possible CC1–SOAR contact site that requires coiled-coil interplays between the predicted Cα1 and Sα4 helices (Fig. 5k,l). Notably, this new site is distinct from the previously reported inhibitory helix spanning the distal regions of CC1 (residues 310–337), which is suggested to interact with the first helix of SOAR (Sα1 helix)^26^,^29^.

## Discussion

To date, a number of studies on STIM1 have been directed exclusively towards either the luminal domain or the cytoplasmic domain (STIM1-CT) (reviewed in refs 5, 15). Although these studies have at large resolved how the STIM1 luminal domain senses Ca^2+^ depletion and how STIM1-CT gates ORAI1 channels, they yielded little or no information regarding how the signal is initially transduced from ER lumen to the cytosol. We recently showed that close apposition of STIM1 at residue 233—the position where the transmembrane domain (STIM1-TM) ends and the cytosolic portion of STIM1 emerges from the ER membrane—could switch STIM1 to adopt an activated conformation to open ORAI1 Ca^2+^ channels^6^,^30^. This points to a potential role of the rearrangement of the single transmembrane domain of STIM1 in propagating the conformational changes towards the cytoplasm. However, mechanistic dissection of the role of STIM1-TM in mediating ER lumen-to-cytosol signal transduction has been hindered due to complications associated with dimerization/oligomerization of both the ER-luminal and the cytoplasmic domains. Here we take advantage of a gain-of-function mutation (that is, C227W) in STIM1-TM to overcome this hurdle. This unique mutant enables us to dissect the activating mechanism of STIM1 by bypassing store depletion and dimerization/oligomerization of the EF-SAM luminal domain^31^,^32^ and the CAD/SOAR domain^9^,^10^. It is generally accepted that CAD/SOAR or other STIM1-CT fragments assemble as a dimer/oligomer regardless of store depletion^9^,^10^,^11^,^13^,^26^,^28^,^29^,^33^, which makes it very challenging to examine the oligomeric state and conformations of the single transmembrane segment *per se* using full-length STIM1. By contrast, C227W allows us to uncouple distinct activation steps and compare the STIM1-TM conformational states without perturbing the structural integrity of other functional components. This gain-of-function mutant seemingly resembles an activated state of full-length STIM1 in several aspects: first, based on our cross-linking and FRET measurement results, C227W brings the N terminus of STIM1-TM in a shorter distance, which likely reflects the impact of Ca^2+^-induced dimerization/oligomerization of EF-SAM on STIM1-TM^31^,^32^; second, both the C227W substitution and store depletion can substantially reduce the CC1–SOAR interaction to similar levels in our FRET assay; third, C227W recapitulates the hallmark physiological responses in the Ca^2+^/NFAT pathways following store depletion. Together, this unique mutant is suited to answer questions that are almost intractable with other gain-of-function mutations found in either the luminal or the cytoplasmic domains of STIM1. For instance, it enables us to compare the resting state and one type of activated state of STIM1-TM with high-resolution NMR and chemical cross-linking, and more importantly, to further address how luminal signals can be transmitted through STIM1-TM to propagate conformational changes throughout the STIM1 cytoplasmic domain. However, we are aware that C227W may not fully mimic the native Ca^2+^-depleted state of STIM1. For example, the resting FRET signal of C227W is slightly higher than Ca^2+^-depleted WT following ionomycin treatment in HEK293 cells co-expressing WT or C227W STIM1_1–237_-CFP/YFP (Fig. 3k,l). In addition, ionomycin evokes a further increase of FRET in C227W, whereas one would expect no significant FRET changes if C227W is identical to the Ca^2+^-depleted state. We speculate that further oligomerization of STIM1, possibly working through the EF-SAM domain or other regions downstream of the SOAR domain, may account for the observed increase in FRET signals upon addition of ionomycin to the mutant C227W.

With a combined use of biochemical, spectroscopic and computational approaches, we have clearly demonstrated that STIM1-TM undergoes structural reorganization when switching to an activated state. Our chemical cross-linking results and computational modelling of STIM1-TM (Supplementary Fig. 3) indicate that the helices of the STIM1-TM dimer interact primarily through the C-terminal positions (residues 221–232), and that a crossing angle of the two helices might separate the lower half of STIM1-TM (residues 214–220) apart. When adopting an activated conformation, it is possible that a reduction of the crossing angle (which is predicted to be over 45° in the computational model of the resting state, Supplementary Fig. 3b) may bring both helical ends closer together. Indeed, we have found that I220 and F229 (or F214 and N234), which are situated below or above the crossing point, exhibit a similar increase in cross-linking (or FRET) efficiency when STIM1-TM assumes an activated conformation.

The molecular determinants that govern STIM1 autoinhibition have been a matter of debate. The idea of STIM1 intramolecular trapping was initially pioneered by Korzeniowski *et al*.^28^, who proposed that an acidic sequence in CC1 (^318^EEELE^322^) could interact with a basic segment of STIM1-CT (^382^KIKKK^386^ in CAD/SOAR). Although the contact sites proposed are in conflict with recently published structural evidence^26^,^29^, the notion of intramolecular switching during STIM1 oligomerization has been well accepted. In two subsequent studies^6^,^18^, both cellular FRET and our *in vitro* LRET assays confirmed the conformational change in activated STIM1 in the context of STIM1_233–474_ (ORAI1-activating small fragment or OASF) or STIM1_233–685_. Mutations predicted to disrupt coiled-coil formations in CC1 (for example, L248S or L251S) switch OSAF or STIM1-CT into extended conformations, pointing to the possibility that the lower region of CC1 is a key molecular determinant mediating STIM1 activation. In the current study, we have further applied a two-component FRET assay to monitor the dynamic CC1–SOAR interaction under physiologically relevant conditions with minimal structural perturbation on CC1 or SOAR. Through a series of carefully designed deletion and mutagenesis studies, we mapped out a minimal region and critical residues that keep SOAR quiescent at rest. In our mapping strategy, we have kept one component (CC1 or SOAR) intact while introducing deletion or point mutations into the other functional domain. Very recently, Fahrner *et al*. have applied a method called FRET-derived interactions in a restricted environment (termed FIRE) to dissect the coiled-coil interplays in STIM1-CT by artificially fusing different short peptide fragments derived CC1 and SOAR to the STIM1 transmembrane helix via a flexible 32-glycine linker^34^. Both studies reached the conclusion that the CC1-α1 helix and the CC3 (or SOAR-α4 helix) are critical for keeping STIM1 quiescent at rest, but they disagreed in the locations of specific CC1–SOAR contact sites. For example, we proposed that L258/L261 are crucial residues in CC1 involved in docking to SOAR while Fahrner *et al*. claimed L251. In our serial deletion study, we clearly show that C-terminal truncation up to L258/L261 or L>G substitution at these two positions substantially weakened the CC1–SOAR interaction. It is anticipated that critical residues identified through the FIRE assay (that is, L251S, two-to-three turns below L258/L261 in an ideal helix) would exhibit similar effects (Supplementary Fig. 7b), because sabotage at the ‘foundation’ of the CC1 core region would certainly damage the SOAR-docking sites located above. Admittedly, since both studies are based on inferences from FRET data without structural validation, we do not exclude the possibilities that other regions in SOAR (for example, predicted CC2, residues 353–388, which has not been examined in our assay) could directly or indirectly contribute to its association with CC1.

The recently published solution structure of a STIM1-CT fragment (residues 312–387) shows two extended helices corresponding to the distal region of CC1 (residues 313–340) with CC2 (equivalent to SOAR-α1 helix) that are connected by a short loop (residues 341–343)^29^. Two U-shaped monomers assemble into a symmetric antiparallel dimer through extensive coiled-coil interactions. The potential interaction between the distal region of CC1 and SOAR is also inferred from the crystal structure of a portion of *Caenorhabditis elegans* STIM1-CT, which shows a segment of CC1 helix docking against the SOAR domain^26^. Deletion of the corresponding segment in human STIM1 resulted in an activated phenotype, thus leading to the proposal that this region (also termed ‘the inhibitory helix’ or the IH domain, corresponding to human STIM1_310–337_) controls the intramolecular conformational switching of STIM1 (refs 26, 35). Nonetheless, the IH domain probably only reflects part of the CC1–SOAR interaction because L251S, L258G/A or L261G substitutions in the initial region of CC1 could overcome this ‘inhibitory’ effect. Very recently, a disease-associated mutant R304W that causes constitutive STIM1 activation has been reported in patients with the Stormorken syndrome^19^. Notably, R304 is located far above the additional SOAR-docking region (233–261) identified through our FRET studies. The introduction of R304W did not affect the interaction between STIM1_1–310_-CFP and YFP-SOAR (Supplementary Fig. 7b), but it significantly weakened the association of STIM1_1–342_-CFP with YFP-SOAR (Supplementary Fig. 7c). Hence, R304 is more likely to perturb the interaction between the distal region of CC1 and SOAR. Indeed, Morin *et al*. proposed a model in which R304W disrupts the electrostatic interaction with E318 and the hydrogen bonding with Q314, thus distorting the IH domain that is also involved in locking SOAR in quiescence^36^. On the basis of these findings, we speculate that the inhibitory helix at the distal region of CC1 seems to be critical for anchoring SOAR (Sα4 helix) and the lower portion of CC1 (Cα1 helix) in a particular orientation that favours their physical contact, and that deletion or structural perturbation of this region would impose structural constraints to prevent SOAR docking towards CC1. However, this hypothesis warrants further test and ultimate structural validation.

**Figure 6:**
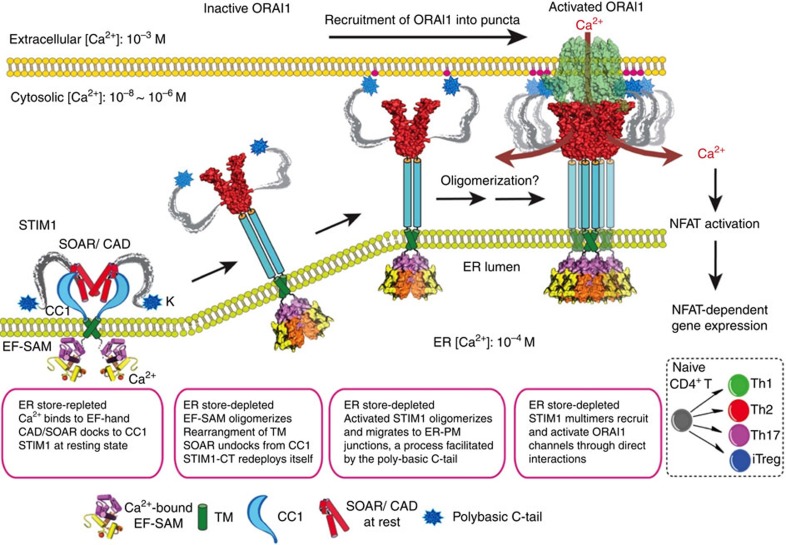
A tentative activation model of SOCE reflecting a STIM1 conformational switch and the dynamic coupling between STIM1 and ORAI1. At a resting condition, the STIM1 luminal EF-SAM domain is loaded with Ca^2+^ and remains largely as a monomer. The STIM1 cytoplasmic domain (STIM1-CT), consisting of a long coiled coil (CC1), a minimal ORAI1-activating region (SOAR or CAD) and a C-terminal polybasic C-tail (K), likely stays as a dimer and adopts a folded-back configuration that keeps itself inactive through the physical interaction between CC1 and SOAR (Cα1:Sα4 and Cα3:Sα1 coiled-coil interplays). Upon ER Ca^2+^ store depletion, dissociation of Ca^2+^ from the EF-SAM domain initiates a destabilization-coupled oligomerization process in the ER lumen. Conformational changes in the canonical EF-hand Ca^2+^-binding motif disrupt the intramolecular interaction between the EF-hands and SAM domains, thereby causing aggregation of the luminal EF-SAM domains. The luminal domain oligomerization further triggers structural rearrangement in the transmembrane domain (STIM1-TM), leading to a closer apposition of the C-terminal end of STIM1-TM. This further propagates conformational changes throughout STIM1-CT by inducing undocking of SOAR from CC1. STIM1-CT redeploys itself and adopts a more extended conformation by exposing the SOAR/CAD domain, as well as the polybasic C-tail. Next, activated STIM1 multimerizes and moves towards the ER–PM junctional sites, where it recruits and directly gates ORAI1 channels possibly through direct physical contacts with both termini of ORAI1. This process is likely facilitated by the interaction between its polybasic C-tail and the negatively charged phosphoinositides (red spheres) in the inner leaflet of the plasma membrane. Sustained Ca^2+^ influx through ORAI1 channels activates downstream effectors such as calcineurin, a Ca^2+^-dependent phosphatase that dephosphorylates the nuclear factor of activated T cells (NFAT) and triggers the nuclear translocation of NFAT to regulate gene expression during lymphocyte activation. Ultimately, activated T cells differentiate into various effector cells, including Th1, Th2, Th17 and iTregs. The relative positioning and orientation of functional domains in activated STIM1 remains unknown. The oligomeric states and structures of the luminal EF-SAM domain and of CAD/SOAR, surface rendered as solid dimers, have not been determined in activated STIM1.

In conclusion, we propose that the single transmembrane domain of STIM1 has a specific role other than to simply connect the luminal and cytoplasmic domains Figure 6. We suggest that it undergoes structural rearrangement during the ER lumen-to-cytosol signal transduction, and that reorganized STIM1-TM prompts a conformation switch in the juxtamembrane CC1 region to release SOAR from contact with a previously unrecognized docking site in CC1. Overall, our multi-pronged approach provides physiologically relevant evidence to support the conformational switch hypothesis during STIM1 activation.

## Methods

### Chemicals and other reagents

Tetramethylrhodamine (TMR)-5-maleimide, fluorescein (FITC)-5-maleimide and α-bungarotoxin (BTX) fluorescent conjugate (Alexa Fluor 555, AF555) were purchased from Life Technologies. Tris(2-carboxyethyl)phosphine (TCEP) was obtained from Pierce (Thermo Scientific). 1,2-dimyristoyl-sn-glycero-3-phosphocholine (DMPC), 1,2-diheptanoyl-sn-glycero-3-phosphocholine (DHPC) and fluorescent lipid 1,2-dioleoyl-sn-glycero-3-phosphoethanolamine-N-(carboxyfluorescein) (PE-FITC) were purchased from Avanti Polar Lipids. Isopropyl-β-D-thiogalactopyranoside (IPTG), copper(II) sulfate, 1,10-phenanthroline, *n*-octyl-β-D-glycoside (OG), lauroylsarcosine sodium, *N*-ethylmaleimide, ionomycin, TG and phorbol 12-myristate 13-acetate (PMA) were purchased from Sigma. Isotopes used for labelling recombinant proteins were from Cambridge Isotope Laboratories. Anti-GFP (sc-8334), anti-YFP (sc-32897, Santa Cruze Biotech Inc.) and anti-CFP (SAB1100419, Sigma) antibodies were used at 1:1,000 dilution. All other reagents were purchased from Sigma-Aldrich.

### Constructs for fluorescence imaging

Full-length complementary DNAs (cDNAs) of human STIM1 was subcloned into pCMV6-XL5 (Origene)^37^ with the insertion of enhanced green fluorescent protein (EGFP), YFP or CFP between two additional NarI sites introduced immediately after residue N39. STIM1 mutant constructs were subsequently made using the QuikChange Lightning site-directed mutagenesis kit (Agilent). For STIM1-CFP and STIM1-YFP constructs, human STIM1 was inserted into pECFP-N1 or pEYFP-N1 between XhoI and BamHI. Truncated STIM1-CFP and STIM1-YFP variants were prepared by standard PCR and ligation. pCDNA3.1(+)-mCherry-ORAI1 was made by inserting mCherry between BamHI and EcoRI restriction sites and human ORAI1 between EcoRI and XhoI sites (Life Technologies). NFAT1_1–460_-GFP^38^, mCherry-CAD^9^, YFP-SOAR^10^ and YFP-D-SOAR (tandem SOAR domain)^39^,^40^ were obtained from Addgene or generated as previously described.

### Constructs for luciferase assays

The full-length cDNA of mouse STIM1 was inserted into pEF4/myc-His B (Life Technologies) between BamHI and XbaI sites for luciferase assays. The luciferase reporter pGL4.30[*luc2P*/NFAT-RE/Hygro] and the pRL-TK plasmid encoding *Renilla* luciferase were purchased from Promega. The luciferase reporter plasmid was further transfected into Jurkat T cells to make a stable reporter cell line (termed Jurkat-NFAT-Luc).

### Constructs for recombinant protein expression in *E. coli*

The sequence of TM-CC1 domain (residues 209–310) was amplified via PCR and cloned into the pProEX HTb vector (Life Technologies) between the BamHI and XhoI sites for expression as (His)_6_-TM-CC1 proteins. In some experiments, the recombinant STIM1 fragments were fused to the B1 domain of streptococcal protein G (GB1), a monomeric small tag that proves to increase the solubility without appreciable perturbation to membrane proteins kept in lipids or membrane mimetics^41^. (His)_6_-TM (residues 209–237) was prepared by introducing a stop codon after residue 237. Their variants, including C227S, C227W and other monocysteine constructs, were made using the QuikChange Lightning site-directed mutagenesis kit (Agilent).

To generate GB1-TM-CC1-LBT-(His)_6_ variants, the cDNA encoding a lanthanide-binding tag (residues GGFIDTNNDGWIEGDELLLEEG)^6^ was inserted downstream of GB1-TM-CC1-His_6_ via a single XhoI site. To produce high-affinity peptide (HAP)-fused proteins, a double-stranded oligo encoding the 13-mer HAP peptide (WRYYESSLLPYPD) was synthesized (Integrated DNA Technologies) and inserted immediately downstream of residue 310 (termed GB1-TM-CC1-HAP-His_6_). The corresponding mutant C227W was generated using the QuikChange Lightning site-directed mutagenesis kit (Agilent).

The plasmid pMCSG9-SOAR^26^ was a gift from Dr Yuequan Shen (Department of Biochemistry and Molecular Biology, Nankai University, Tianjin, China) and the plasmid pMSP1D1 used to make the membrane scaffold protein for nanodiscs was obtained from Dr Stephen Sligar (Department of Chemistry, University of Illinois Urbana-Champaign, Urbana, IL).

### Real-time intracellular Ca^2+^ measurements

Intracellular Ca^2+^ levels were measured with Fura-2 AM by following our previous procedures^13^,^14^,^40^,^42^. In brief, HEK ORAI1-CFP stable cells cultured on coverslips were kept in a dye-loading solution (107 mM NaCl, 7.2 mM KCl, 1.2 mM MgCl_2_, 1 mM CaCl_2_, 11.5 mM glucose and 20 mM HEPES–NaOH (pH 7.2)) with 2 μM Fura-2 AM for 30 min. Cells were then kept in Fura-2 AM free solution for another 30 min. For cells transfected with STIM1-activating mutations, 300 μM Ca^2+^ or nominally Ca^2+^-free solution were used to keep cells healthy. Fura-2 signals were recorded using a ZEISS oberserver-A1 microscope equipped with a Lambda DG4 light source (Sutter Instruments), Brightline filter sets (part number: FURA2-C-000, Semrock Inc.), a × 40 oil objective (numerical aperture=1.30) and an iXon3 EMCCD camera (Oxford Instruments), and the MetaFluor software (Molecular Devices). Emission fluorescence at 505 nm generated by 340 nm excitation light (*F*_340_) and 380 nm light (*F*_380_) was collected every 2 s, and intracellular Ca^2+^ levels are shown as *F*_340_/*F*_380_ ratio. All experiments were carried out at room temperature. Traces shown are representative of at least three independent repeats with each including 30–60 single cells.

### Epifluorescence imaging and FRET measurements

The same system used in Ca^2+^ measurements plus an Optosplit II Image Splitter (Cairn Research Limited) was used for FRET measurements. CFP (428.9±5.5_Ex_/465±32_Em_), YFP (502.6±11.2_Ex_/549±21_Em_) and FRET_raw_ (428.9±5.5_Ex_/549±21_Em_) filters were used to capture images (F_CFP_, F_YFP_ and F_raw_, respectively) every 10 s at room temperature. Three-channel-corrected FRET was calculated as previously described^40^,^42^,^43^. FRET signal was calculated using the following formula: FRET_c_=*F*_raw_−*F*_d_/*D*_d_ × *F*_CFP_−*F*_a_/*D*_a_ × *F*_YFP_, where FRET_c_ represents the corrected total amount of energy transfer, *F*_d_/*D*_d_ represents measured bleed-through of CFP into the FRET filter (0.826) and *F*_a_/*D*_a_ represents measured bleed-through of YFP through the FRET filter (0.048). To reduce variations caused by differences in expression levels, FRET_c_ values were normalized against donor fluorescence (*F*_CFP_) to generate an N-FRET (normalized FRET) signal. To eliminate instrument-dependent factors, apparent FRET efficiency, *E*_app_, was calculated using the following equation: *E*_app_=N-FRET/(N-FRET+G)^43^, where G (4.59) is the system-dependent factor. It is obtained with partial YFP photobleaching method: G=(FRET_c_−FRET_c_^post^)/(F_CFP_^post^−F_CFP_), where FRET_c_^post^ and F_CFP_^post^ are corresponding FRET_c_ and F_CFP_ values after partial photobleach of YFP^43^. The intensity of the light used to bleach YFP was carefully chosen so that it will not bleach CFP at the same time. All fluorescence images were collected and briefly processed with MetaFluor software (Molecular Devices), and then the resulting data were further analysed with Matlab R2012b software and plotted with Prism5 software. Representative traces of at least three independent experiments performed on 15–30 cells are shown as mean±s.e.m.

### Electrophysiological measurements

Whole-cell recordings were performed using transfected HEK293 cells. Briefly, currents were recorded using an EPC-10 Mac-driven patch-clamp amplifier (HEKA Elektronik). All recordings were performed at room temperature. Command voltage protocol generation and data acquisition were performed using Patchmaster (HEKA Elektronik). The membrane potential was held at 0 mV, and 190-ms voltage ramps from −100 to 90 mV were delivered every 2 s. The standard Cs^+^-containing pipette solution consisted of the following (mM): 130 Cs glutamate, 8 NaCl, 0.9 CaCl_2_, 12 EGTA and 10 HEPES; pH=7.3, adjusted with CsOH. This solution was supplemented with 10 mM MgCl_2_ to inhibit the endogenous Mg^2+^-inhibited cation (MIC/TRPM7) channels. The standard extracellular solution contained (mM) the following: 150 Na aspartate, 2 CaCl_2_, 2 MgCl_2_, 4.5 KCl and 10 HEPES; pH=7.3, adjusted with NaOH. The cell capacitance and pipette capacitance were compensated during recording using the software; series resistance was not compensated. In Fig. 1e–g, the time courses were not leak-subtracted. The current–voltage (*I*–*V*) relationships were corrected for leakage; the residual currents after 10 μM Gd^3+^ treatment were considered as the leak. The HEK cells we used have residual endogenous TRPM7 currents^44^, which might account for the outward currents shown in our leak-subtracted, break-in *I*–*V* plot. Data were analysed with OriginPro 8 software (OriginLab) and are expressed as mean±s.e.m.

### Confocal and TIRF imaging

Cell lines used for imaging include HEK293 and HeLa cells. All cells were grown in DMEM (Sigma) supplemented with 10 mM HEPES and 10% heat-inactivated fetal bovine serum, unless otherwise noted. Transfections were performed using Lipofectamine 2000 (Life Technologies) following the manufacturer’s protocol. To aid efficient and stable puncta formation, the DMEM medium was substituted by pre-warmed Ca^2+^-free Hank’s balanced salt solution before imaging. TG (0.5–1 μM) or 2.5 μM ionomycin was used to induce store depletion. Live cell imaging was performed at room temperature with × 60 oil lens on an inverted Nikon Eclipse Ti-E microscope customized with A1R-A1 confocal and motorized total internal reflection fluorescence (TIRF) modules using argon-ion (405 and 488 nm) and helium-neon (543 nm) or diode (561 nm) as laser sources. In some experiments, a Zeiss LSM 700 confocal system equipped with a × 100 oil lens (numerical aperture=1.45) was used to obtain images with higher resolution. Image analysis was performed using the NIS-Elements software (Nikon) or Image J (NIH).

### NFAT-related assays

For the NFAT nuclear translocation assay, a HeLa cell line stably expressing NFAT_1–460_-GFP was established to monitor the cellular localization of NFAT. Cells were cultured on glass-bottom dishes (MatTek), transfected with pMCV6-XL5-CFP-STIM1 (WT or C227W) constructs and imaged 24 h post transfection.

For NFAT-responsive luciferase assay, HEK293T cells were cultured in 96-well plates and transfected with pEF4/myc-His B-STIM1 (WT or C227W) constructs and NFAT-luciferase reporter gene pGL4.30[*luc2P*/NFAT-RE/Hygro] (Promega). The *Renilla* luciferase gene (pRL-TK) was also co-transfected as a control for counting transfected cells and calculating transfection efficiency. Jurkat-NFAT-Luc stable cells were electroporated with pEF4/myc-His B-mSTIM1 (WT or C227W) plasmids using the Neon transfection system (Life Technologies). Transfected cells were aliquoted in a 24-well plate in RPMI 1640 medium supplemented with 10% FBS. At 16–18 h after transfection, both HEK293T and Jurkat cells were treated with trace amounts of dimethyl sulfoxide solution (as mock), PMA (1 μM) or PMA+TG (1 μM). Cells were harvested after an additional 8 h. Luciferase activity was assayed using the Dual Luciferase Reporter Assay System (Promega) on a Biotek Synergy2 luminescence microplate reader. The ratio of firefly to *Renilla* luciferase activity was plotted for HEK293T cells and the results for Jurkat cells were represented as firefly luciferase activity. All the data were normalized against the mock group.

### Recombinant protein expression and purification

*Escherichia coli* strain BL21 (DE3) cells (EMD Millipore) were transformed with plasmids encoding various STIM1 constructs, and grown at 37 °C in LB medium with 100 mg l^−1^ of ampicillin. Protein expression was induced by the addition of 500 μM IPTG when OD_600_ of the culture reached 0.6–0.8, followed by incubation for another 3–4 h. Harvested cells were resuspended in a resuspension buffer containing 20 mM Tris-HCl pH 7.4, 10 mM imidazole, 200 mM NaCl, 2 mM TCEP and 0.6% *N*-lauroylsarcosine or 1% OG, and sonicated. The cellular debris was removed by centrifugation. In the case of His_6_-tagged proteins, the lysate was applied to Ni^2+^-nitrilotriacetic acid (Ni-NTA)-agarose resin (Qiagen). Bound recombinant proteins were eluted in 20 mM Tris pH 7.4, 250 mM imidazole, 150 mM NaCl, 1 mM TCEP and 0.6% *N*-lauroylsarcosine or 1% OG and further purified by gel filtration on a Superose 6 10/300 GL column or Superdex 200 10/300 GL column (GE Healthcare).

### Nuclear magnetic resonance spectroscopy

His_6_-STIM1-TM (WT and C227W) proteins were reconstituted into bicelles for NMR studies. For expression of stable isotope-labelled proteins, a 5-ml culture was initially grown in LB media at 37 °C overnight. The cells were gently pelleted and resuspended into 500 ml M9 minimal media. The media was supplemented with ^15^N-NH_4_Cl, ^13^C-glucose, ^13^C/^2^H-glucose or D_2_O (Cambridge Isotope Laboratories), according to standard labelling schemes. Cultures were further grown until an OD_600_ of 0.6 before inducing protein expression with 1 mM IPTG at 37 °C for 4 h and then were harvested by centrifugation. Cell pellets were resuspended in a buffer containing 20 mM Tris pH 8.0, 200 mM NaCl, 2 mM TCEP and sonicated. His_6_-TM-CC1 was isolated in an inclusion body in the absence of appropriate detergents. The supernatant and inclusion body were separated by centrifugation. The inclusion body was resuspended by 20 mM Tris-HCl pH 7.4, 10 mM imidazole, 200 mM NaCl, 2 mM TCEP and 8 M urea. The lysate was applied to Ni-NTA resin (Qiagen). Bound recombinant proteins were eluted in 20 mM Tris pH 7.4, 250 mM imidazole, 150 mM NaCl, 1 mM TCEP and 8 M urea. The eluted protein was dialysed to ddH_2_O to remove urea. The precipitates were centrifuged and dissolved by TFE in a round-bottom flask mixed with DMPC and DHPC lipids (Avanti Polar Lipids). A protein to DMPC ratio of 1:100 and a *q*-factor of 0.25 (the DMPC/DHPC molar ratio) were generally applied. The mixture was dried to a thin film under an N_2_ stream followed by high-speed vacuum overnight. The dried film was resuspended in a sample buffer solution (20 mM Tris, pH 7.2, 200 mM NaCl and 5 mM TCEP) and was subjected to repeated cycles of freeze and thaw until a clear solution was obtained.

All NMR spectra were acquired at 27 °C on Bruker Avance 600 and 850-MHz spectrometers. The acquired data were further processed using the software package NMRPipe^45^ and analysed with Sparky^46^.

### Fast protein liquid chromatography

Size-exclusion chromatography coupled with multi-angle laser light scattering measurements were performed on a GE FPLC system with a Superdex 200 10/300 GL column (GE Healthcare) and an in-line eighteen-angle DAWN HELLOS II instrument equipped with an Optilab rEX Refractive Index Detector (Wyatt Technology) by following previous procedures^6^,^14^. Five hundred microlitres of 10–100 μM protein samples were subjected to chromatography in a buffer consisting of 20 mM Tris−HCl pH 7.3, 150 mM NaCl, 2 mM dithiothreitol and 0.6% *N*-lauroylsarcosine at a flow rate of 0.3 ml min^−1^. The protein concentrations were calculated from the absorbance at 280 nm, and the light scattering data were collected at 663 nm. Molecular weight was calculated using the ASTRA software (Wyatt Technology).

### Pull-down assay to probe CC1–SOAR interaction *in vitro*

In all, 100 μl of 1 mg ml^−1^ of MBP (used as negative control) or MBP-SOAR was immobilized on 100 μl amylose resin (New England Biolabs), and incubated for 4 h at 4 °C with each 400 μg of the recombinant CC1 variants in 1 ml buffer containing 20 mM Tris pH 7.5, 150 mM NaCl and 1 mM TCEP (TN buffer), followed by 10-time washing with the TN buffer to eliminate nonspecific binding. The resin was mixed with 100 μl TN buffer and 4 × SDS gel-loading buffer, heated at 100 °C for 10 min and briefly centrifuged prior to gel electrophoresis. Samples were separated on 16% SDS–PAGE. Bound proteins were visualized on SDS–PAGE after Coomassie Brilliant Blue R-250 staining.

### Copper/phenanthroline-catalysed disulfide cross-linking

CuP-catalysed disulfide cross-linking studies were carried out as described previously^13^. In brief, 1 mM CuSO_4_/3 mM 1,10-phenanthroline was added to 50 μl of purified recombinant proteins (1 mg ml^−1^) in 20 mM HEPES pH 7.4, 150 mM NaCl and 1 mM TCEP. The mixture was incubated on ice for 15 min or for the time indicated. Reactions were stopped by the addition of an equal volume of quenching solution consisting of 20 mM HEPES, 150 mM NaCl, 100 mM *N*-ethylmaleimide and 50 mM EDTA, pH 7.4. Samples were mixed with 5 × non-reducing SDS loading buffer, heated at 60 °C for 10 min and subjected to electrophoresis on 15% SDS–PAGE or 8–16% NuPAGE (Life Technologies). Each cross-linking experiment was repeated independently for at least three times. The disulfide cross-linking efficiency for each cysteine substitution was determined as the ratio of dimeric GB1-TM-CC1 to total GB1-TM-CC1 (monomer plus dimer). Quantification of the fraction of GB1-TM-CC1 was performed using the program Image J (NIH).

### Protein conjugation with dyes

Monocysteine-containing TM-CC1 (on the background of C227S and C227W) variants were labelled with FITC-maleimide and TMR-maleimide for FRET measurements. In all, 10–20 mM dye stocks were prepared in anhydrous DMF immediately prior to use. A total of 50∼100 μM protein samples in 20 mM Tris-HCl pH 7.3, 150 mM NaCl and 1% OG were first treated with 10 mM TCEP to completely break the disulfide bonds. Prior to the reaction with dye, excess TCEP was removed by quickly passing the sample through a PD-10 desalting column (GE Healthcare) and the buffer was exchanged to PBS, 1 mM TCEP and 1% OG. The reactive dye (20-fold molar excess) was added into 50 μM of reduced protein sample in a total volume of ∼500 μl with constant stirring. The mixture was incubated at room temperature for 2 h or at 4 °C overnight with constant agitation. Next, 100 mM 2-mercaptoethanol was added to the mixture to stop the reaction. After labelling, free dye was removed by first passing the sample through Ni-NTA resin and then through a PD-10 column (GE Healthcare). Efficiently labelled protein yielded a ∼0.8–0.9:1 molar ratio of fluorophore to protein, based on measuring the absorbance at 492 nm (FITC maleimide, *ε*=83,000 M^−1^ cm^−1^), 541 nm (TMR maleimide, *ε*=95,000 M^−1^ cm^−1^; Molecular Probes Handbook, 11th edition) and 280 nm in 6 M guanidine HCl (Pierce) for the protein.

### Nanodisc assembly

*E. coli* strain BL21(DE3) cells (EMD Millipore) were transformed with plasmids encoding membrane scaffold protein 1D1 (MSP1D1) and grown at 37 °C in LB medium with 50 mg l^−1^ of kanamycin. MSP1D1 purification and nanodisc assembly were performed as previously described with slight modifications^6^. A fluorescent lipid, 1,2-dioleoyl-*sn*-glycero-3-phosphoethanolamine-*N*-(carboxyfluorescein) (PE-FITC) is sparsely incorporated into the nanodisc to track the assembly of nanodiscs, and more importantly, is utilized as a donor in the FRET assay or acceptor in the LRET assay to examine the distance between the C-terminal end of assayed recombinant proteins and the lipid bilayer in nanodisc. In brief, MSP1D1 at 0.1–0.2 mM concentration was combined with cholate-solubilized DMPC and the fluorescent lipid PE-FITC to reach a final molar ratio of 1:65:0.5. After incubation at room temperature for 2 h, the self-assembly process is initiated by incubation with adsorbent Bio-beads M2 (Bio-Rad) for at least 2 h. Nanodiscs incorporating GB1-TM-CC1-HAP (WT and C227W) or GB1-TM-CC1-LBT (WT and C227W) were assembled with the MSP1D1:DMPC:target protein:PE-FITC ratio of 1:65:0.5:0.5. Assembled TM-CC1 nanodiscs were analysed by gel filtration on a Superose 6 10 300^−1^ GL column (GE healthcare). Successful incorporation of TM-CC1 was further confirmed by SDS–PAGE and fluorescence measurement for each fraction. The plotted fluorescence trace representing incorporated TM-CC1 overlaid well with the ultraviolet trace (absorbance of MSP1D1 and TM-CC1) of the assembled nanodiscs (Supplementary Fig. 4).

### Luminescence resonance energy transfer assay

LRET assays were performed on the QuantaMaster 40 spectrofluorometer equipped with a pulsed xenon excitation source for phosphorescence lifetime measurement^6^. In brief, a Tb^3+^:protein ratio of 0.9:1 was adopted. The spectra were collected from 450 to 650 nm with the excitation set at 280 nm and the slit widths set at 4–6 nm. When acquiring the luminescence spectra, a gate of 200 μs was set to eliminate fluorescence arising from directly excited acceptor and scattered light.

### Circular dichroism spectroscopy

Circular dichroism spectra of recombinant proteins were recorded in a Jasco-715 spectropolarimeter at 22 °C using a 1-mm path length quartz cell with the protein concentration at 15–20 μM in PBS containing 1% OG at pH 7.3. All spectra were obtained as the average of at least eight scans with a scan rate of 50 nm min^−1^. The ellipticity was measured from 190 to 260 nm and converted to mean residue molar ellipticity (deg cm^2^ dmol^−1^ res^−1^).

### Fluorescence spectroscopy

Fluorescence spectra were acquired on a QM40 spectrofluorometer (Photon Technology International). To measure inter-subunit FRET in the context of TM-CC1 with C227S or C227W as background, FITC- or TMR-labelled monocysteine variants were assayed in PBS, pH 7.3 containing 1% OG and 1 mM TCEP. The fluorescence spectra were recorded from 490 to 650 nm with the excitation set at 475 nm to minimize the directly excited emission from the acceptor. To enable the FRET measurements between the C terminus of TM-CC1-HAP and the fluorescent nanodisc, Alexa Fluor 555-labelled β-BTX was mixed with TM-CC1-HAP (residual BTX removed through gel filtration) to form a stable acceptor complex^6^,^47^. The fluorescence spectra were recorded from 490 to 650 nm with the excitation at 475 nm in 20 mM Tris-HCl, pH 7.3, 150 mM NaCl and 2 mM TCEP.

### Biacore surface plasmon resonance measurements

Biacore surface plasmon resonance experiments were performed at 25 ° C on a Biacore 3000 (GE Healthcare Bio-Sciences AB, Uppsala, Sweden). CC1 sensor surface was prepared using ligand thiol coupling, with the cysteine residue at the N terminus of CGG-CC1(233–342) (ref. 6). The immobilization was performed on a CM3 sensor chip at 5 μl min^−1^ flow rate with PBS (10 mM sodium phosphate pH 7.4, 150 mM NaCl). Flow cell was activated with 15 μl of EDC/NHS followed by 30 μl PDEA (GE Healthcare) to introduce a reactive disulfide group on the surface. Thirty microlitre of CC1 (10 μg ml^−1^ in 10 mM sodium acetate pH 5.5) was injected onto the activated surface and then blocked with 30 μl of cysteine-NaCl. Approximately 650 response units of CC1 were immobilized. A reference flow cell was prepared with activation and blocking steps, but without any protein coupled. Binding study was carried out at 50 μl min^−1^ flow rate using TBS (20 mM Tris, pH 7.0, 150 mM NaCl) containing 0.1% BSA (w/v) as running buffer. MBP-SOAR (0–16 μM, 2 × dilution) or MBP stock in TBS was first diluted twofold in TBS containing 0.2% BSA, then further diluted with running buffer and injected onto CC1 and reference surfaces. Background-corrected sensorgrams were collected and analysed using BIAevaluation (version 4.1).

### Gel electrophoresis with native PAGE

Native PAGE was performed using 4–16% NativePAGE Novex Bis-Tris gels (Life Technologies). Samples were prepared using the NativePAGE Sample Prep Kit (Life Technologies) following the manufacturer’s protocols. Samples and unstained protein standards (NativeMark, Life Technologies) were run in cold anode (50 mM BisTris, 50 mM Tricine, pH 6.8) and deep-blue cathode buffers (50 mM BisTris, 50 mM Tricine, pH 6.8 and 0.02% Coomassie G-250) for 1 h at 150 V, whereupon the cathode buffer was replaced with slightly blue cathode buffer (50 mM BisTris, 50 mM Tricine, pH 6.8, 0.02% Coomassie G-250) and electrophoresis continued for 1–2 h at 180 V. Following electrophoresis, gels were stained with Coomassie brilliant blue R-250 or transferred to polyvinylidene difluoride membranes for western blotting.

### Computational modelling of STIM1-TM

The structure of STIM1-TM was predicted with CATM^48^. CATM is a program for the structural prediction of GAS_right_ motifs, a common structural motif for transmembrane dimerization characterized by GxxxG and similar sequence patterns formed by small amino acids separated (Gly, Ala and Ser) at *i*, *i*+4 (ref. 24). GxxxG-like motifs are frequently involved in transmembrane association^24^, where they allow the formation of networks of hydrogen bonds between Cα–H donors and carbonyl oxygen acceptors on opposed helices^19^. The sequence of STIM1-TM contains two GxxxG-like motifs (S_219_xxxG_223_ and G_226_xxxA_230_). CATM is distributed with the MSL software library v. 1.2 (ref. 49), available at http://msl-libraries.org.

### Statistical analyses

Unless otherwise noted, quantitative data are expressed as the mean and s.e.m. Statistical significance was determined with a two-tailed Student’s *t*-test. *P<0.05; **P<0.01; ***P<0.001.

## Additional information

**How to cite this article:** Ma, G. *et al*. Inside-out Ca^2+^ signalling prompted by STIM1 conformational switch. *Nat. Commun*. 6:7826 doi: 10.1038/ncomms8826 (2015).

## Supplementary Material

Supplementary Information Supplementary Figures 1-11, Supplementary Table 1 and Supplementary References

Supplementary Movie 1 Real-time TIRF imaging of YFP-SOAR in HEK293-ORAI1 cells co-expressing WT (left) or C227W (right) STIM1-1-310-CFP before and after store depletion induced by ionomycin.

Supplementary Movie 2 Real-time confocal imaging of ionomycin-induced dissociation of YFP-SOAR (left) from STIM1-1-310-CFP (right) in HEK293 cells.

Supplementary Movie 3 Real-time confocal imaging of YFP-SOAR (left) in HEK293 cells co-expressing STIM1-1-310-C227W-CFP (right).

Supplementary Movie 4 Real-time confocal imaging of ionomycin-induced dissociation of mCherry-CAD (left) from STIM1-1-265-CFP (right) in HEK293 cells.
