# Supplementary material for: Inside-out Ca2+ signalling prompted by STIM1 conformational switch
Source: Nat Commun. 2015 Jul 17;6:7826. doi: 10.1038/ncomms8826 (PMC4509486; doi:10.1038/ncomms8826)
Supplement: Supplementary Information — Supplementary Figures 1-11, Supplementary Table 1 and Supplementary References [file ncomms8826-s1.pdf]

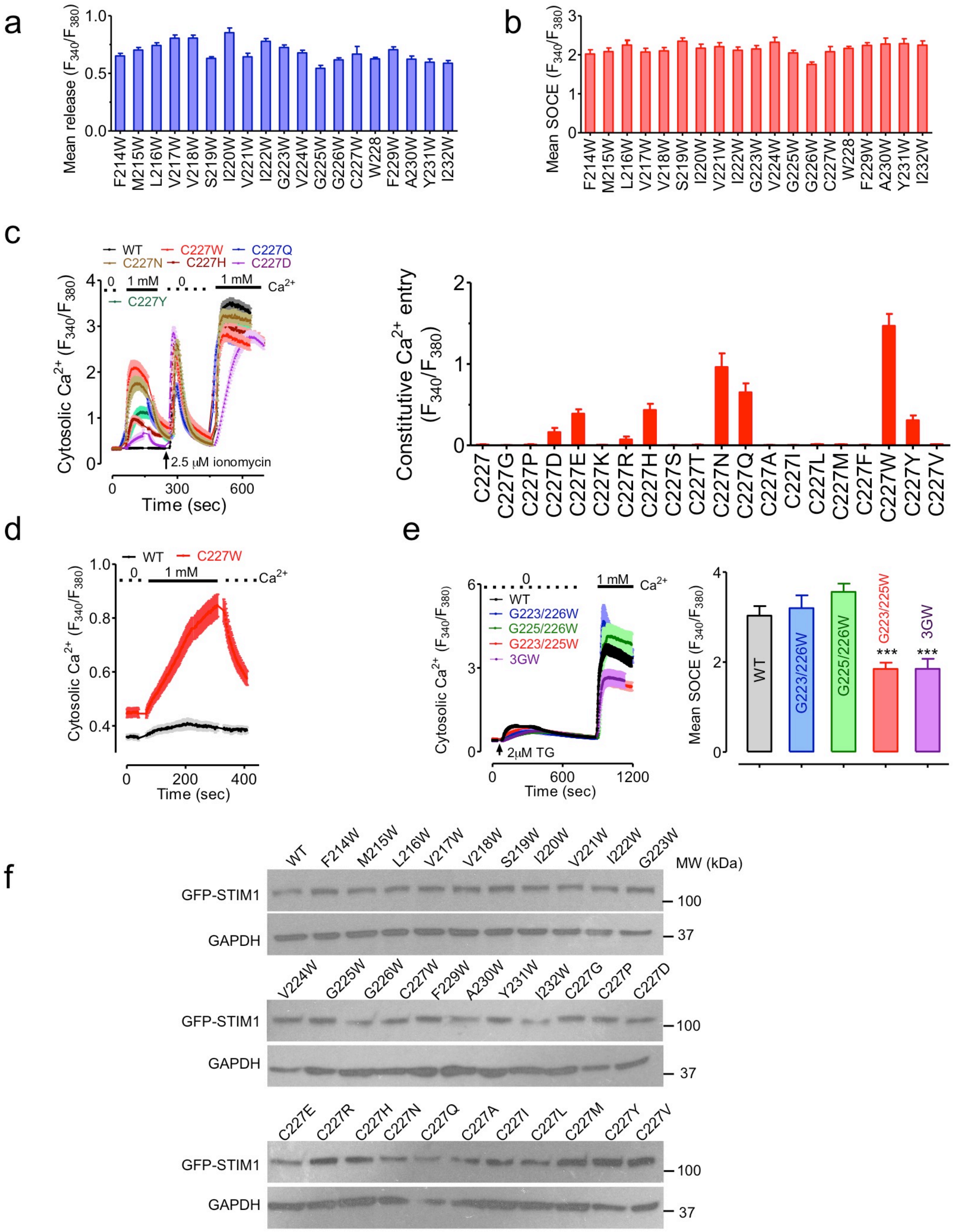
a
Mean release (F_{340}/F_{380})
F214W M215W L216W V217W V218W S219W I220W V221W I222W G223W V224W G225W G226W C227W W228 F229W A230W Y231W I232W
b
Mean SOCE (F_{340}/F_{380})
c
WT C227W C227Q C227N C227H C227D C227Y
0 1 mM 0 1 mM Ca2+
Cytosolic Ca2+ (F_{340}/F_{380})
2.5 μM ionomycin
Time (sec)
Constitutive Ca2+ entry (F_{340}/F_{380})
C227 C227G C227P C227D C227E C227K C227R C227H C227S C227T C227N C227Q C227A C227I C227L C227M C227F C227W C227Y C227V
d
WT C227W
Time (sec)
e
WT G223/226W G225/226W G223/225W 3GW
2μM TG
Mean SOCE (F_{340}/F_{380})
f
WT F214W M215W L216W V217W V218W S219W I220W V221W I222W G223W MW (kDa)
GFP-STIM1 100
GAPDH 37
V224W G225W G226W C227W F229W A230W Y231W I232W C227G C227P C227D
C227E C227R C227H C227N C227Q C227A C227I C227L C227M C227Y C227V

Supplementary Figure 1. Ca^{2+} influx reported by ratiometric Fura-2 fluorescence in HEK293-ORAI1 stable cells or native HEK293 cells expressing mutant STIM1T-TM constructs.

a-b, Effects of tryptophan substitution of STIM1-TM residues on ER calcium release (first peak in Ca^{2+} response curve, **a**) and SOCE (second peak, **b**).

c, *Left,* Ca^{2+} influx in HEK293-ORAI1 stable cells expressing WT and STIM1-C227X mutants monitored by Fura-2 fluorescence ratio. Store depletion was induced by 2.5 μM ionomycin. The solid line above the curves indicates 1 mM Ca^{2+} in the external medium. *Right,* Effects of substitution of C227 by other 19 amino acids on constitutive Ca^{2+} influx when switching the external medium from 0 to 1 mM Ca^{2+} without store depletion. In addition to C227W, gain-of-function phenotypes were observed in negatively-charged variants, C227D and C227E, as well as in several other substitutions (C227H, C227N, C227Q, C227Y) that might promote the formation of hydrogen bonds in the side chains.

d, Ca^{2+} influx in HEK293 cells expressing WT or C227W STIM1 constructs monitored by Fura-2 fluorescence ratio. The level of constitutive Ca^{2+} entry was quantified as the difference of the mean Fura-2 fluorescence ratio between the peak value in the presence of 1 mM Ca^{2+} and the basal value without externally-added Ca^{2+}. Store depletion was induced by 2 μM TG.

e, Ca^{2+} influx in HEK293-ORAI1 cells expressing WT and G>W STIM1 mutants monitored by Fura-2 fluorescence ratio. Store depletion was induced by 2.5 μM ionomycin. The solid line above the curves indicates 1 mM Ca^{2+} in the external medium.

f, Detection of the expression of EGFP-STIM1 variants used in the Ca^{2+} flux assay by anti-GFP immunoblotting. ***P < 0.001 when compared to WT (two-tailed Student's t-test) in panel e. All the error bars denote s.e.m. for 30-60 cells assayed in three independent experiments.

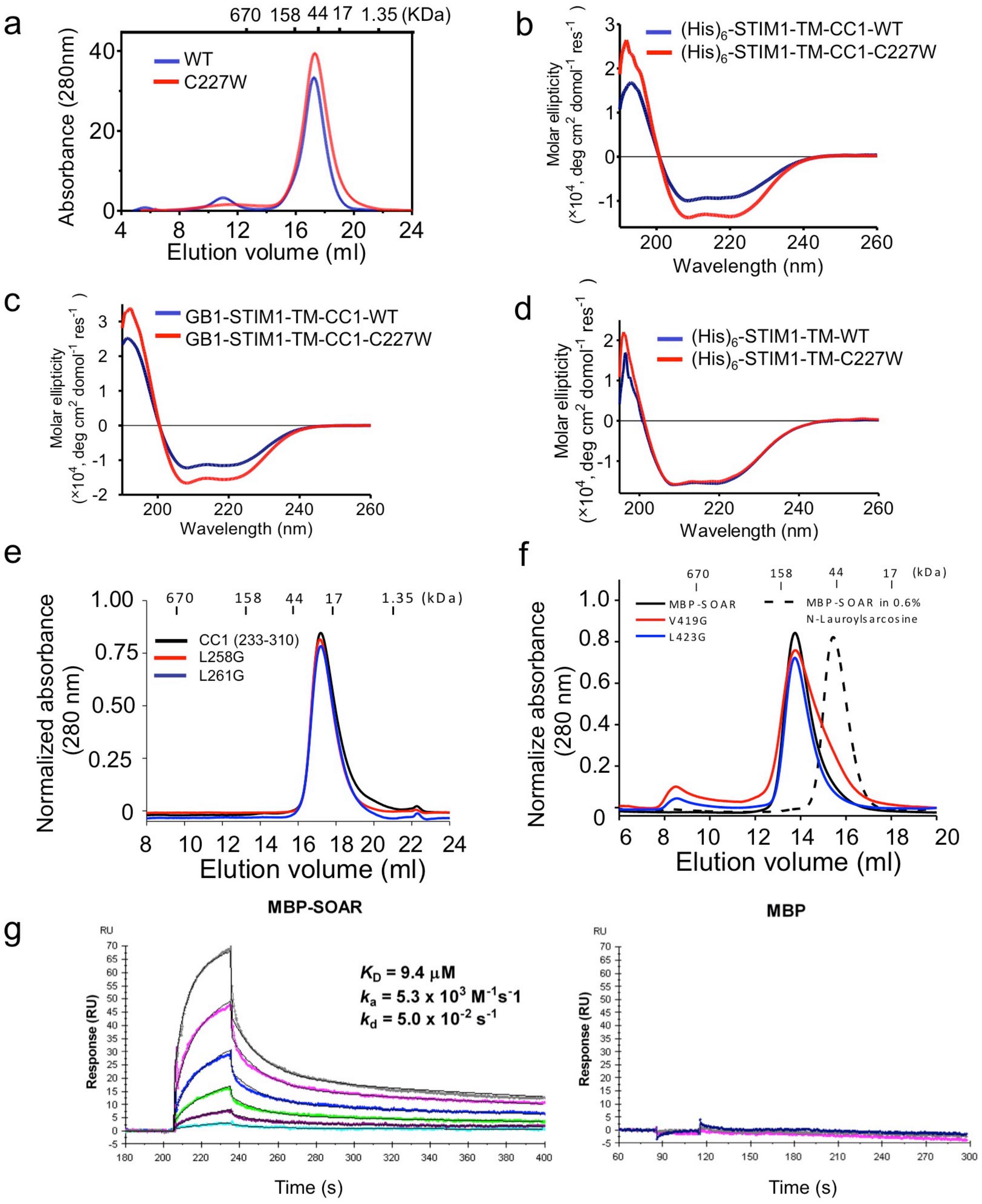
a
670 158 44 17 1.35 (KDa)
WT
C227W
Absorbance (280nm)
Elution volume (ml)
b
(His)6-STIM1-TM-CC1-WT
(His)6-STIM1-TM-CC1-C227W
Molar ellipticity
(×10^4, deg cm^2 domol^-1 res^-1)
Wavelength (nm)
c
GB1-STIM1-TM-CC1-WT
GB1-STIM1-TM-CC1-C227W
Molar ellipticity
(×10^4, deg cm^2 domol^-1 res^-1)
Wavelength (nm)
d
(His)6-STIM1-TM-WT
(His)6-STIM1-TM-C227W
Molar ellipticity
(×10^4, deg cm^2 domol^-1 res^-1)
Wavelength (nm)
e
670 158 44 17 1.35 (kDa)
CC1 (233-310)
L258G
L261G
Normalized absorbance (280 nm)
Elution volume (ml)
f
670 158 44 17 (kDa)
MBP-SOAR
V419G
L423G
MBP-SOAR in 0.6% N-Lauroylsarcosine
Normalize absorbance (280 nm)
Elution volume (ml)
g
MBP-SOAR
RU
Response (RU)
K_D = 9.4 μM
k_a = 5.3 x 10^3 M^-1s^-1
k_d = 5.0 x 10^-2 s^-1
Time (s)
MBP
RU
Response (RU)
Time (s)

Supplementary Figure 2. *In vitro* characterization of recombinant STIM1 proteins.

a, Gel filtration elution profiles of $(His)_6$-STIM1-TM-CC1 (residues 209-310) proteins. Both WT (blue) and C227W (red) were eluted at the similar elution volume, implying no significant change in the oligomeric state.

b, Far-UV CD spectra of recombinant $(His)_6$-STIM1-TM-CC1 proteins. The gain-of-function mutant C227W assumed more negative ellipticity.

c, Far-UV CD spectra of recombinant GB1-STIM1-TM-CC1 proteins. Compared to WT (blue), C227W (red) adopted more helical content given its higher negative ellipticity. GB1, immunoglobulin-binding B1 domain of *streptococcal* protein G.

d, Far-UV CD spectra of recombinant $(His)_6$-STIM1-TM (residues 209-237) proteins. No significant difference in ellipticity was observed between WT (blue) and C227W (red) STIM1-TM.

e, Gel filtration elution profiles of $(His)_6$-STIM1-CC1 (residues 233-310; WT, black; L258G, red; L261G, blue). All proteins were largely eluted at the similar elution volume, implying no significant change in the oligomeric state.

f, Gel filtration elution profiles of MBP-SOAR variants (residues 344-444, theoretical MW: 55.8 kDa; WT, black; V419G, red; L423G, blue). All proteins in the absence of detergents were eluted at comparable elution volumes, implying no significant change in the oligomeric state. The apparent molecular weight (120 kDa) is comparable to the size of a dimer. Notably, MBP-SOAR was eluted at a size comparable to monomer (estimated MW: 50 kDa) in the presence of detergent (0.6% N-laurylsarcosine, dashed black line).

g, Surface plasmon resonance (SPR) measurement of the interaction between MBP-SOAR and immobilized CC1(233-342). SPR response curves for MBP-SOAR (twofold serial dilution from 16 to 0. 5 μM) were shown in colored lines. The sensorgrams were globally fitted to a bivalent analyte binding model (fitted lines shown in black). Kinetic constants were obtained from curve fitting (Chi^2 = 0.18) and used for calculating the dissociation constant K_D ($K_D = k_d/k_a$). The binding model was selected on the basis of closeness of fit and the expectation that purified dimeric SOAR binds two identical CC1 subunits. MBP (8, 16 and 32 μM), used as negative control in the assay, did not bind to the sensor chip with immobilized CC1.

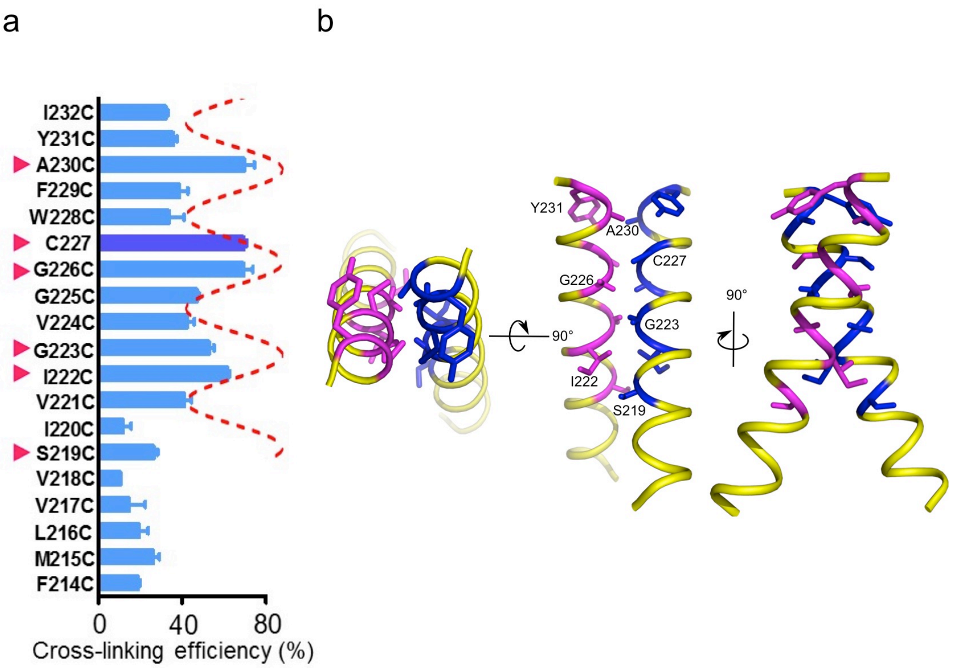

Supplementary Figure 3. CuP-catalyzed cross-linking of STIM1-TM monocysteine variants.

a, Shown is the quantification of cross-linking efficiency of STIM1-TM monocysteine variants in the cysteine-less background of C227S. The helical repeat pattern was imposed above the bar graph as a red dashed line. Most of the monocysteine variants in the upper half of STIM1-TM exhibits a basal crosslinking efficiency of ~30-40% under our crosslinking condition, presumably due to the relatively high rotational mobility of the protein backbone or closer apposition of this region in isolated STIM1-TM under our assay conditions *in vitro*. Notably, the crosslinking efficiency in the lower half of STIM1-TM was significantly reduced, likely reflecting the high rigidity of protein backbone from residues F214 to I220 or the N-terminal region splaying apart from each other. Error bars denote s.e.m. for three independent experiments.

b, The cross-linking data is consistent with a structural model obtained *ab initio* using the program CATM[1], which predicts that the inactive conformation of STIM1-TM is compatible with the conformation of a GAS_{right} dimer mediated by an extended GxxxG-like interaction motif ($S_{219}xxxG_{223}xxxC_{227}$)[2]. The dimer is shown from three orientations and the amino acids that interact at the helix-helix interface are highlighted in magenta and blue in the two helices. The same interfacial positions are also marked with an arrow in panel **a**. There is an excellent correspondence between the predicted interface and the positions with the highest level of cross-linkling, whereas the low level of cross-linking in the N-terminal (bottom) region may be explained by the increased distance of the helices, which cross in the C-terminal side of the dimer (top).

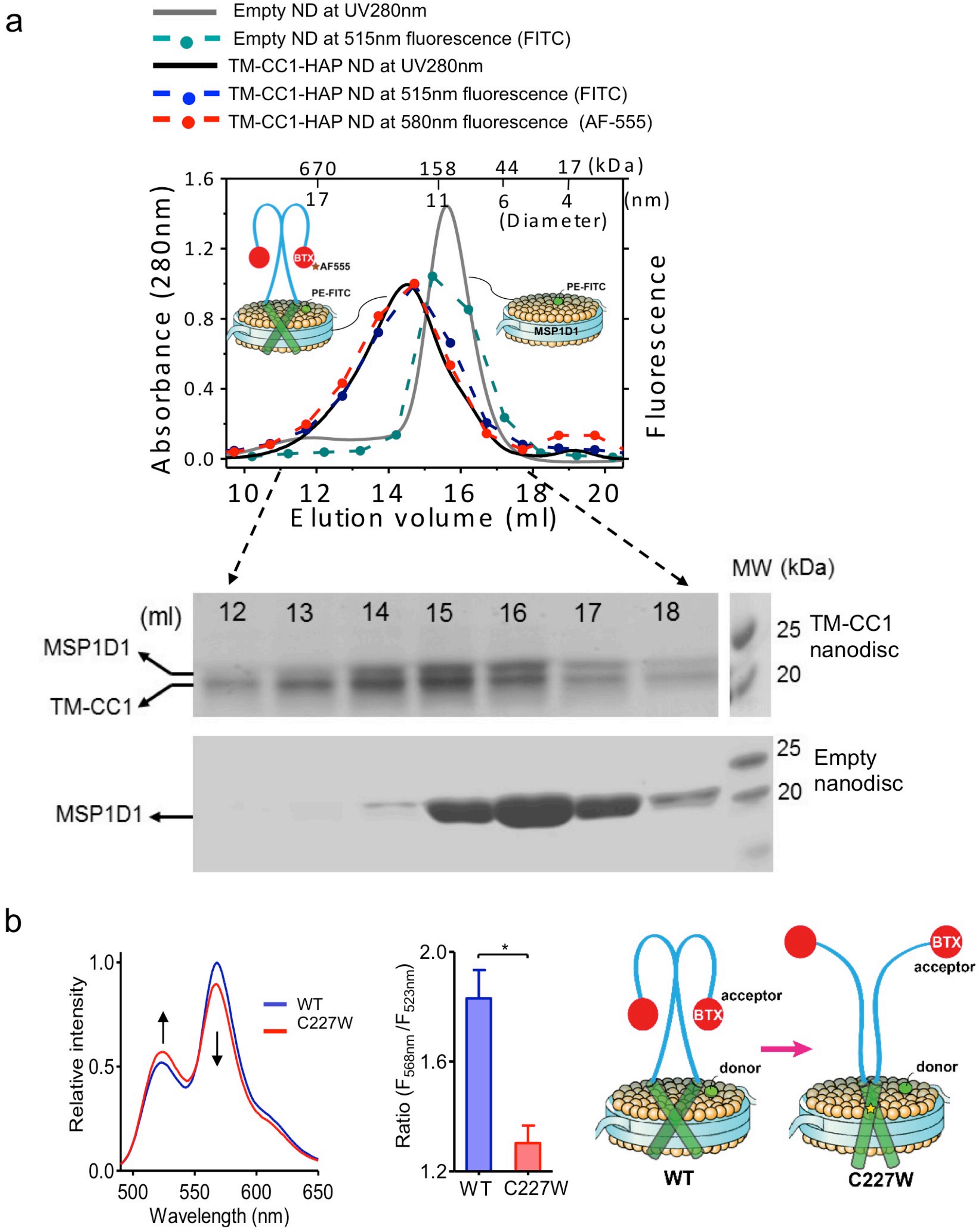
a
Empty ND at UV280nm
Empty ND at 515nm fluorescence (FITC)
TM-CC1-HAP ND at UV280nm
TM-CC1-HAP ND at 515nm fluorescence (FITC)
TM-CC1-HAP ND at 580nm fluorescence (AF-555)
670 158 44 17 (kDa)
17 11 6 4 (nm)
(Diameter)
BTX
AF555
PE-FITC
MSP1D1
Absorbance (280nm)
Fluorescence
Elution volume (ml)
MW (kDa)
(ml) 12 13 14 15 16 17 18
MSP1D1
TM-CC1
25 20
TM-CC1 nanodisc
Empty nanodisc
b
Relative intensity
Wavelength (nm)
WT
C227W
Ratio (F568nm /F523nm)
acceptor
donor
WT
C227W

Supplementary Figure 4. Assembly of TM-CC1 nanodiscs (NDs) and FRET measurements.

a, *In vitro* characterization of assembled NDs. *Upper panel,* overlay of elution profiles representing STIM1 TM-CC1-HAP and empty nanodiscs. A symmetric elution peak centered at ~14.5 ml, as well as the co-migration of FITC-conjugated fluorescent lipid and fluorescent bungarotoxin (BTX-AF555), indicated the assembly of homogeneous TM-CC1 nanodiscs. The fluorescent lipid (λ_{emi} = 523 nm) was used to trace the assembled nanodiscs. TM-CC1 with its C-terminus fused to a 13-mer HAP peptide can readily form a tight docking site for bungarotoxin (BTX) conjugated with acceptor fluorophore, Alexa Fluor 555 (AF-555). Thus, BTX-AF555 (λ_{emi} = 580 nm) was used to report the elution of the TM-CC1-HAP-BTX complex. *Inset,* cartoons of the assembled NDs. *Lower panel*, SDS-PAGE analysis of assembled TM-CC1 or empty nanodiscs. Co-migration of TM-CC1-HAP with the membrane scaffold protein (MSP) further confirmed the assembly of TM-CC1 nanodiscs.

b, FRET measurements were made on TM-CC1 nanodisc with its C-terminus fused to a 13-mer HAP2 peptide, forming a tight docking site for bungarotoxin (BTX) conjugated with acceptor fluorophore, Alexa Fluor 555. A green fluorescent phospholipid analog incorporated into the nanodisc serves as donor. *Left*, Corrected fluorescence spectra of labeled WT (blue) or C227W (red) TM-CC1 nanodiscs (λ_{exc} = 475 nm). *Middle*, quantification of fluorescence ratio at acceptor (568 nm) and donor (523 nm) emission peaks. *Bottom*, cartoon interpreting the results. The relative position of TM helices and the solution structure of CC1 have not been determined. **P* < 0.05 (n=5, paired Student's *t*-test). Error bars denote s.e.m. for five independent experiments.

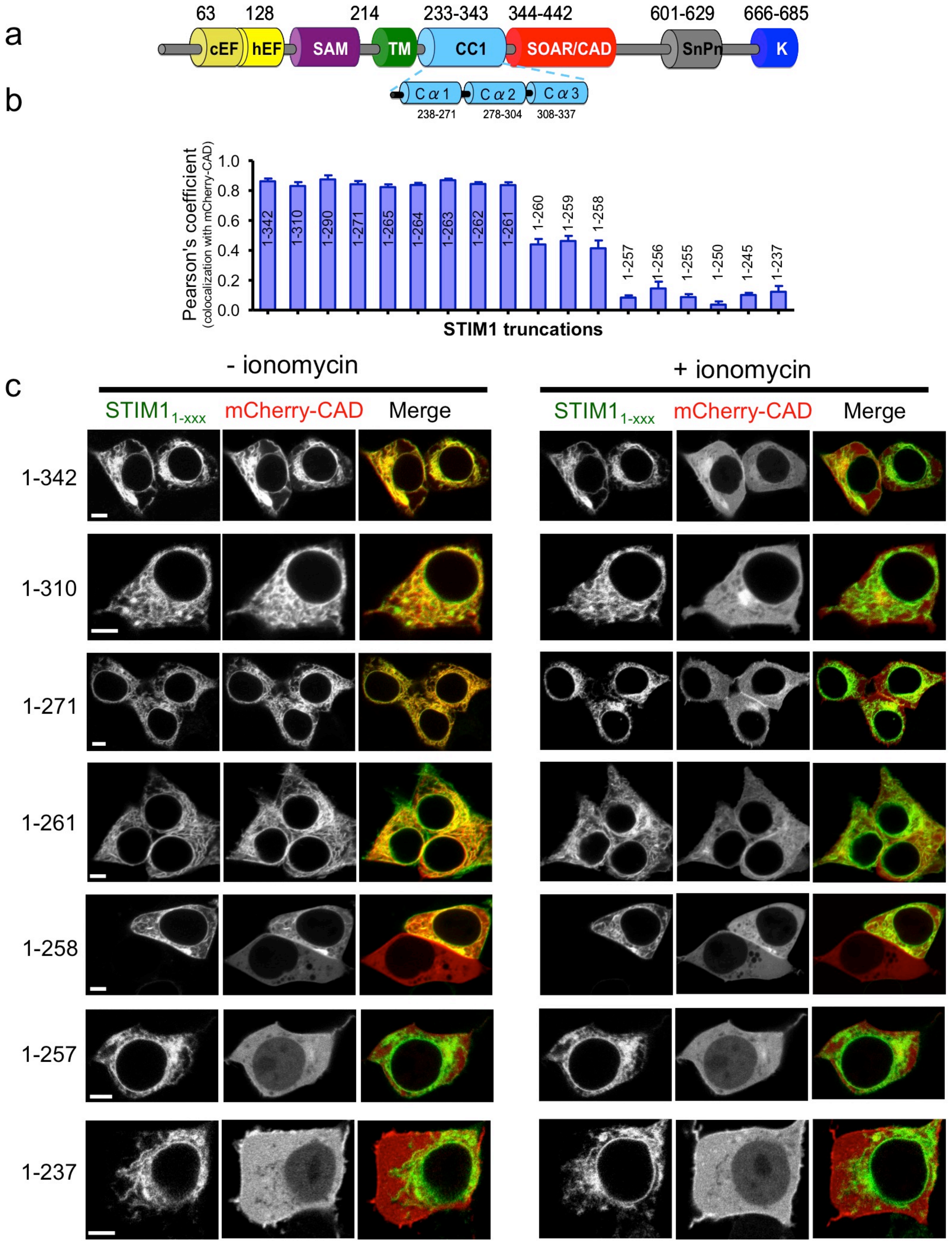
a
63
128
214
233-343
344-442
601-629
666-685
cEF
hEF
SAM
TM
CC1
SOAR/CAD
SnPn
K
b
Cα1
Cα2
Cα3
238-271
278-304
308-337
Pearson's coefficient
(colocalization with mCherry-CAD)
STIM1 truncations
1-342
1-310
1-290
1-271
1-265
1-264
1-263
1-262
1-261
1-260
1-259
1-258
1-257
1-256
1-255
1-250
1-245
1-237
c
- ionomycin
+ ionomycin
STIM1 1-xxx
mCherry-CAD
Merge
1-342
1-310
1-271
1-261
1-258
1-257
1-237

Supplementary Figure 5. Mapping SOAR/CAD-docking sites in CC1.

a, Domain architecture of STIM1-CC1. CC1 was predicted to have three potential coiled coil regions (Cα1-Cα3). A series of C-terminally truncated STIM1-CC1 variants (truncated from residue 342 till residue 237) was generated to test their colocalizaiton with mCherry-CAD. The same set of CFP-tagged constructs was used to examine their interaction with YFP-SOAR/CAD by FRET (**Fig. 3d**).

b, Quantitative analysis on the strength of CC1-SOAR interaction by Pearson's correlation coefficients derived from colocalization assays (mCherry-CAD + $STIM1_{1\text{-}342\text{-}}$CFP variants). **c,** Representative confocal images of HEK293 cells co-expressing mCherry-CAD and truncated $STIM1_{1\text{-}342}$-CFP variants. 2.5 μM ionomycin was added to trigger store depletion. In merged panels, CFP signals were shown as green to aid better visualization. Scale bar, 5 μm.

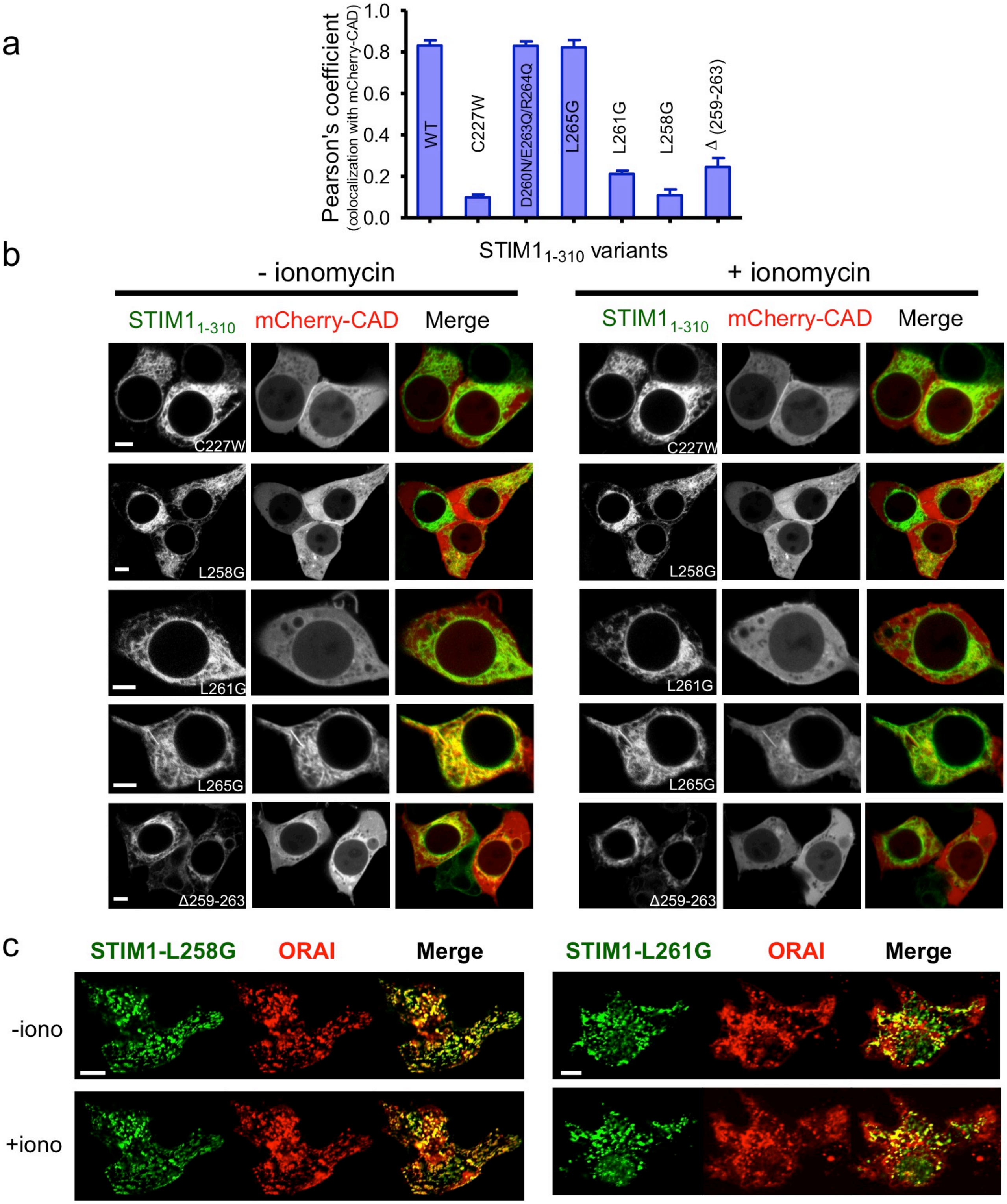
a
Pearson's coefficient
(colocalization with mCherry-CAD)
1.0
0.8
0.6
0.4
0.2
0.0
WT
C227W
D260N/E263Q/R264Q
L265G
L261G
L258G
Δ (259-263)
STIM1 1-310 variants
b
- ionomycin
+ ionomycin
STIM1 1-310
mCherry-CAD
Merge
C227W
L258G
L261G
L265G
Δ259-263
c
STIM1-L258G
ORAI
Merge
STIM1-L261G
-iono
+iono

Supplementary Figure 6. Identifying critical residues involved in CC1-CAD interaction by colocalization assay.

a, Quantitative analysis of colocalization of STIM1-1-310-CFP mutants with mCherry-CAD by Pearson correlation coefficient. Mutations neutralizing the charges (D260N/E263Q/R264Q) did not disrupt the docking of mCherry-CAD to STIM1-1-310-CFP. On the contrary, mutations perturbing the coiled coil formation (L258G or L261G), along with the deletion of residues 259-263, substantially reduced CC1-SOAR colocalization. The same set of CFP-tagged constructs was used to examine their interaction with YFP-SOAR by FRET (**Fig. 3e**).

b, Representative confocal images of HEK293 cells co-expressing mCherry-CAD and truncated or mutated STIM1-1-310-CFP variants. 2.5 μM ionomycin was added to trigger store depletion. In merged images, CFP and mCherry signals were shown in green and red, respectively, to aid better visualization. Scale bar, 5 μm. **c,** Confocal images of HEK293 cells co-expressing mCherry-ORAI1 (red) and full-length mutant STIM1 constructs (L258G and L261G, green) before and after store depletion induced by 2.5 μM ionomycin (iono). Scale bar, 5 μm.

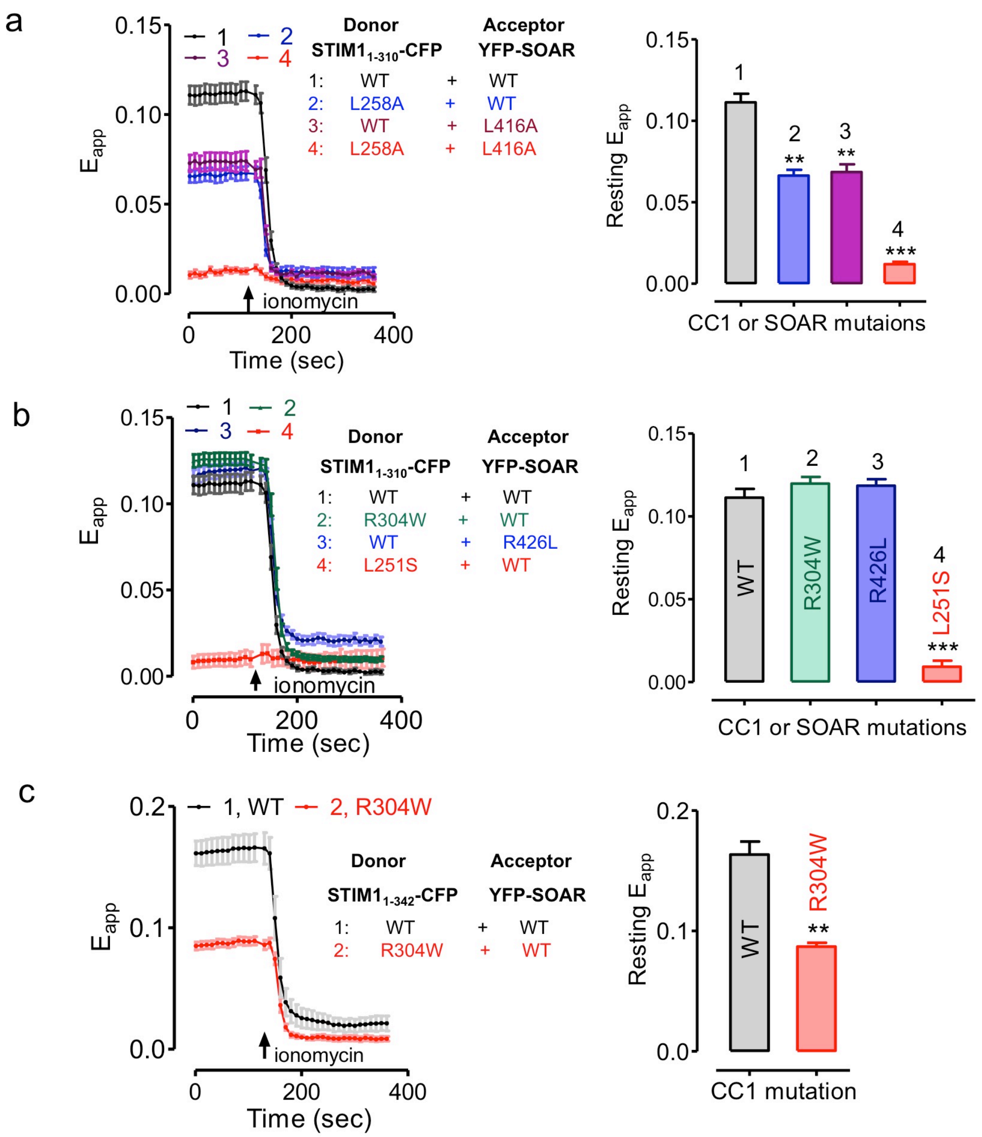

Supplementary Figure 7. Effects of indicated gain-of-function or loss-of-function mutations on the CC1-SOAR interaction.

a-b, Real-time FRET signals in HEK293 cells co-expressing WT or mutant STIM1$_{1-310}$-CFP with YFP-SOAR. Panel **a**, leucine-to-alanine mutants; panel **b**, L251S and R426L described by Fahrner *et al* and the mutant R304W reported by Nesin *et al*[3].

c, Real time FRET signals monitored in HEK293 cells co-expressing WT or R304W STIM1$_{1-342}$-CFP with YFP-SOAR. The resting FRET signals were plotted as bar graph on the right. Error bars denote s.e.m. for at least three independent experiments representing 10-15 individual cells. $^{**}P < 0.01$, $^{***}P < 0.001$ when compared to WT (two-tailed Student's t-test) in panels. All error bars denote s.e.m. for at least three independent experiments.

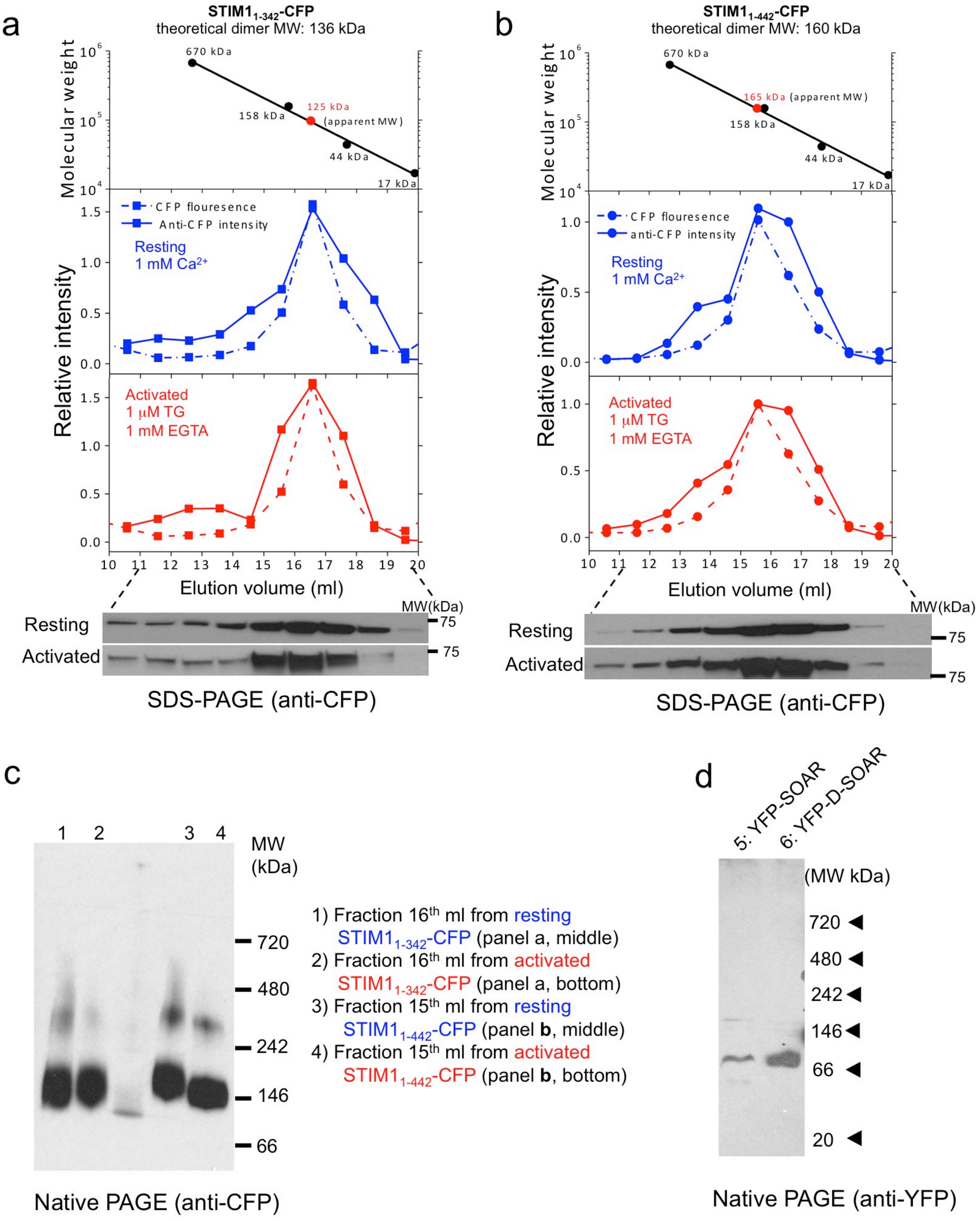
a
STIM1 1-342-CFP
theoretical dimer MW: 136 kDa
Molecular weight
670 kDa
158 kDa
125 kDa
(apparent MW)
44 kDa
17 kDa
CFP flouresence
Anti-CFP intensity
Resting
1 mM Ca2+
Activated
1 μM TG
1 mM EGTA
Relative intensity
Elution volume (ml)
MW(kDa)
Resting
Activated
75
SDS-PAGE (anti-CFP)
b
STIM1 1-442-CFP
theoretical dimer MW: 160 kDa
Molecular weight
670 kDa
165 kDa (apparent MW)
158 kDa
44 kDa
17 kDa
CFP flouresence
anti-CFP intensity
Resting
1 mM Ca2+
Activated
1 μM TG
1 mM EGTA
Relative intensity
Elution volume (ml)
MW(kDa)
Resting
Activated
75
SDS-PAGE (anti-CFP)
c
1 2 3 4
MW (kDa)
720
480
242
146
66
1) Fraction 16th ml from resting STIM1 1-342-CFP (panel a, middle)
2) Fraction 16th ml from activated STIM1 1-342-CFP (panel a, bottom)
3) Fraction 15th ml from resting STIM1 1-442-CFP (panel b, middle)
4) Fraction 15th ml from activated STIM1 1-442-CFP (panel b, bottom)
Native PAGE (anti-CFP)
d
5: YFP-SOAR
6: YFP-D-SOAR
(MW kDa)
720
480
242
146
66
20
Native PAGE (anti-YFP)

Supplementary Figure 8. Characterization of 1-342-CFP, 1-442-CFP, YFP-SOAR and YFP-D-SOAR *in vitro*.

a-b, Plot of FPLC protein standards (black, top panel), inferred molecular weight of protein samples (red, top panel), CFP fluorescence signal from the elution (dashed line, middle and bottom panels), and anti-CFP intensities (solid line, quantified from WB blots) for each fraction against gel filtration elution volume. The cell lysate of HEK293 cell overexpressing $STIM1_{1\text{-}342}$-CFP (a) or $STIM1_{1\text{-}442}$-CFP (b) was solubilized in cell lysis buffer containing 0.6% N-lauroylsarcosine and then subjected to gel filtration with 20 mM Tris buffer pH 7.4, 150 mM NaCl, 1 mM TCEP, 0.6% N-lauroylsarcosine for fractionization. The protein was detected by anti-CFP immunoblotting, with the protein level in each fraction quantified by ImageJ. Both proteins were eluted with the peak elution volume approximately matching the size of dimer. To mimicking store depletion, 1 mM EGTA and 1 μM TG were added to HEK293 cells prior to cell lysis and 1 mM EDTA was applied throughout the experiments to chelae Ca^{2+}. The resting condition was maintained by adding 1 mM extra Ca^{2+} in the buffer.

c-d, Separation of indicated protein samples on reducing native PAGE. Lysates from HEK293 cells expressing $STIM1_{1\text{-}342}$-CFP ($MW_{theoretical}$ = 68 kDa, lanes 1-2, panel **c**), $STIM1_{1\text{-}442}$-CFP ($MW_{theoretical}$ = 80 kDa, lanes 3-4, panel **c**), YFP-SOAR ($MW_{theoretical}$ = 39 kDa, lane 5, panel **d**) or YFP-D-SOAR ($MW_{theoretical}$ = 52 kDa, lane 6, panel **d**) were applied onto native PAGE and then subjected to gel electrophoresis at 4 °C. $STIM1_{1\text{-}342}$-CFP, $STIM1_{1\text{-}442}$-CFP and YFP-SOAR/CAD predominantly migrated as dimer on native PAGE, whereas YFP-D-SOAR stayed as monomer.

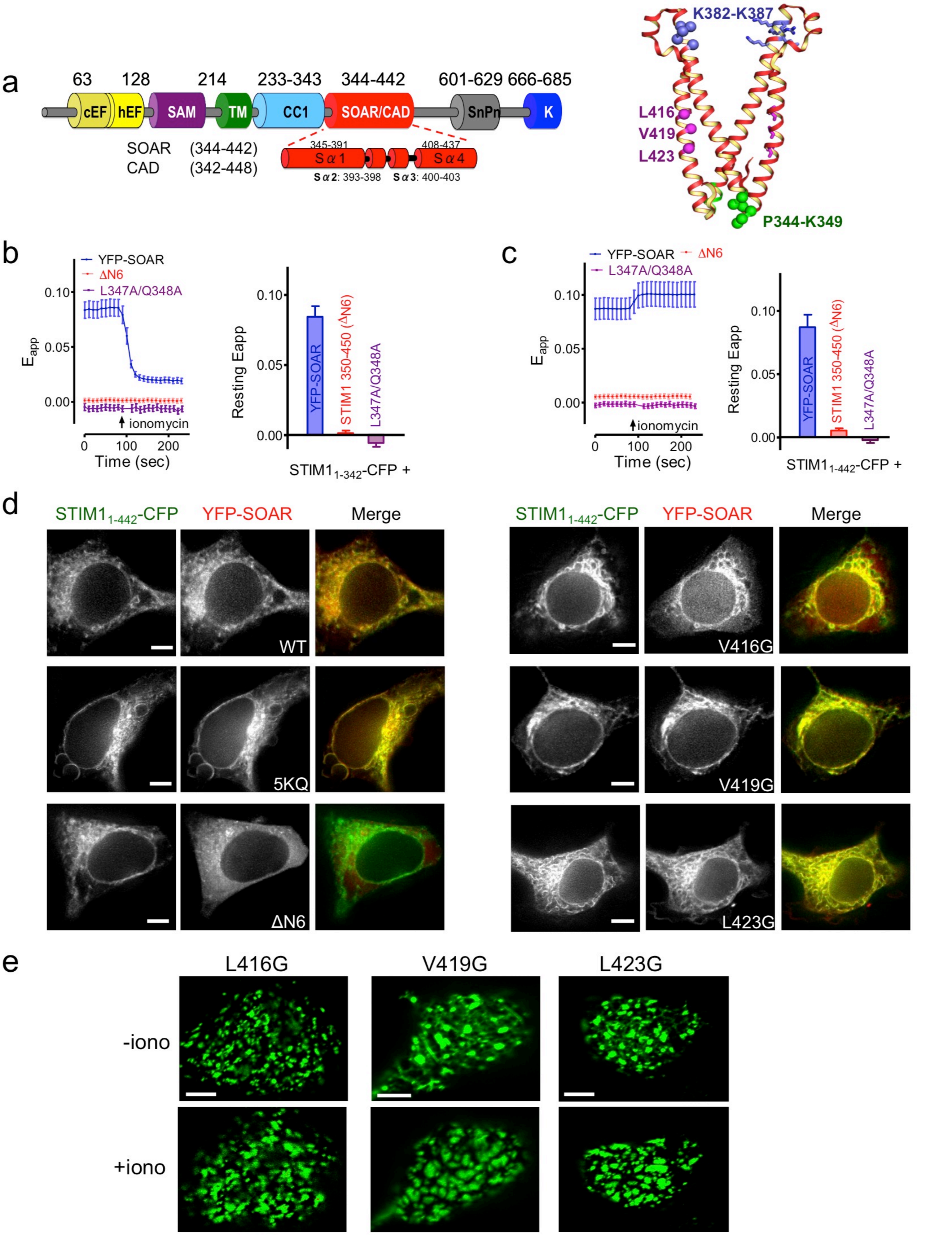

a
63 128 214 233-343 344-442 601-629 666-685
cEF hEF SAM TM CC1 SOAR/CAD SnPn K
SOAR (344-442)
CAD (342-448)
345-391 408-437
Sα1 Sα4
Sα2: 393-398 Sα3: 400-403
K382-K387
L416
V419
L423
P344-K349
b
YFP-SOAR
ΔN6
L347A/Q348A
E_app
ionomycin
Time (sec)
Resting Eapp
YFP-SOAR
STIM1 350-450 (ΔN6)
L347A/Q348A
STIM1_1-342-CFP +
c
YFP-SOAR
ΔN6
L347A/Q348A
E_app
ionomycin
Time (sec)
Resting Eapp
STIM1_1-442-CFP +
d
STIM1_1-442-CFP
YFP-SOAR
Merge
WT
5KQ
ΔN6
V416G
V419G
L423G
e
L416G
V419G
L423G
-iono
+iono

Supplementary Figure 9. Pinpointing CC1-interacting sites within SOAR.

a, Domain architecture and 3-D structure of SOAR. The SOAR crystal structure contains four helices named successively as Sα1-Sα4 (PDB entry: 3TEQ). Key regions participating in SOAR dimerization (344-349, green), and potential residues involved in association with STIM1-CC1 (L416, V419, and L423, magenta) or ORAI1 (382-387, blue) were highlighted.

b-c, Deletion of residues 344-349 (termed ΔN6, red line) or substitution of L347/Q348 with A347/A348 (LQ>AA, purple line) in YFP-SOAR abolished its interaction with STIM1-1-342-CFP (**b**) and also disrupted its capability to form heterodimer with the other SOAR fragment in the context of STIM1-1-442 (**c**). FRET signals were monitored before and after ionomycin-induced store depletion in HEK293 cells. The resting FRET signals were plotted as bar graph. Notably, residues 344-349 were located at the SOAR dimerization contact interface.

d, Representative fluorescence images of HEK293 cells co-expressing STIM1-1-442-CFP and YFP-SOAR variants. In the merged panels, CFP and YFP signals were shown as green and red, respectively, to aid better visualization. Scale bar, 5 μm.

e, Confocal images of the footprint of HEK293 cells expressing full-length STIM1 constructs with indicated mutations in the SOAR domain before and after store depletion induced by 2.5 μM ionomycin (iono). Scale bar, 5 μm.

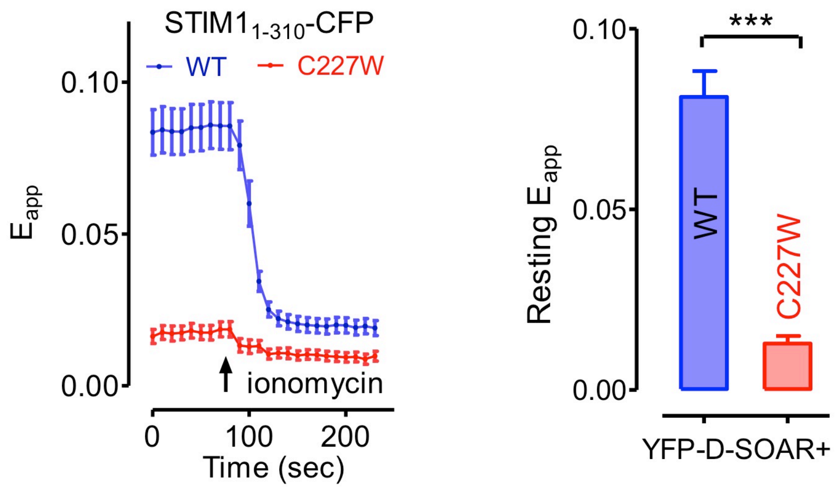

Supplementary Figure 10. Real-time FRET signals monitored before and after ionomycin-induced store depletion in HEK293 cells co-expressing YFP-D-SOAR and STIM1$_{1\text{-}310}$-CFP WT or C227W. ***P < 0.001 (two-tailed Student's *t*-test), Error bars denote s.e.m. for at least three independent experiments representing 10-20 cells.

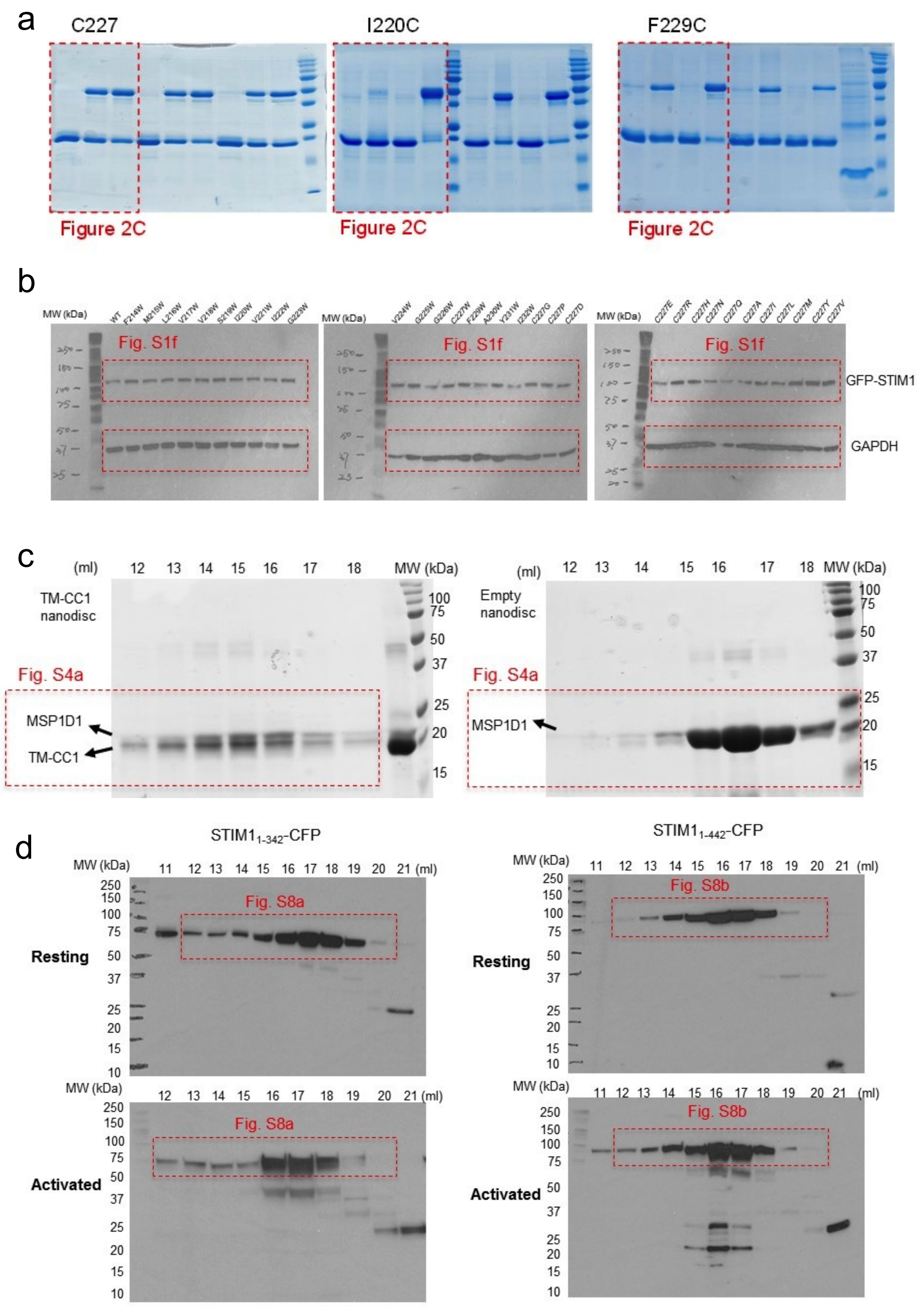

Supplementary Figure 11. Uncropped western blots or SDS-PAGE.

a, uncropped SDS-Page of **Figure 2c**; **b,** uncropped western blots of **Fig. S1f**; **c**, uncropped SDS-page of **Fig. 4a**; **d**, uncropped western blots of **Fig. S8a and Fig. S8b.** The red dished rectangles represent panels shown in the noted figures.

Supplementary Table 1. Oligonucleotide primers used in this study

Primer Name	Sequence (5'-3')	Note
STIM1_XhoI_for	CCG CTCGAG ATG GAT GTG TGC GCC CGT CTT G	For insertion into pECFP-N1, pEYFP-N1, pECFP-C1 or pEYFP-N1
STIM1_BamHI_rev	CCG GGATCC CTT CTT AAG AGG CTT CTT AAA AAT TTT G	
STIM1_209_XhoI_for	CCG CTCGAG AATCAC CTCAAGGACTTCATG	
STIM1_237_BamHI_rev	CCG GGATCC CCCGAACCTCCGTAACGGTTCTGGATATAGGC	
STIM1_310_ BamHI_rev	CCG GGATCC CCCGAACCTCCCTCATTCTCAGTACCCTCCCT	
STIM1_342_ BamHI_rev	CCGGGATCCCCCGAACCTCCATACCATGAGCTGTGTGATTC	
STIM1_442_ BamHI_rev	CCGGGATCCCCCGAACCTCCGACAATCTGGAAACCGCAGAG	
STIM1-BamHI-For	CGG GGATCC ATGGATGTGTGCGCC CGTCTTGCC	For insertion into pEF4/myc-His B
STIM1_xbaI_rev	CCG TCTAGA CTT CTT AAG AGG CTT CTT AAA AAT TTT	
STIM1_Forward	GGAGGTTCGGGG GATCCACCG	For generation of truncated variants of STIM1$_{1\text{-}310}$-CFP
STIM1_290_rev	GATCTCATCTCGCAGCTTCTT	
STIM1_271_rev	CTCCTCCTGGGCCTTGTGCAG	
STIM1_265_rev	CAGCCTTTCCTGAAGGTCATG	
STIM1_264_rev	CCTTTCCTGAAGGTCATGCAG	
STIM1-263_rev	TTCCTGAAGGTCATGCAGACT	
STIM1_262_rev	CTGAAGGTCATGCAGACTCTG	
STIM1_261_rev	AAGGTCATGCAGACTCTGCTC	
STIM1_260_rev	GTCATGCAGACTCTGCTCAGC	
STIM1_259_rev	ATGCAGACTCTGCTCAGCCCG	
STIM1_258_rev	CAGACTCTGCTCAGCCCGGTG	
STIM1_257_rev	ACTCTGCTCAGCCCGGTGTAA	
STIM1_256_rev	CTGCTCAGCCCGGTGTAACCC	
STIM1_255_rev	CTCAGCCCGGTGTAACCCTTC	
STIM1_250_rev	CCCTTCCAGATCCTTCATCAT	
STIM1_245_rev	CATCATTTTCTTCATGTGCTC	
mcherry_ORAI_EcoRI_for	CCG CCG GAA TTC ATG CAT CCG GAG CCC GCC C	For insertion into pCDNA3.1(+)-mCherry
mcherry_ORAI_XhoI_rev	CCG CCG CTC GAG *CTA* GGC ATA GTG GCT GCC GG	
STIM1_209_BamHI_for	CGG GGATCC AATCAC CTCAAGGACTTCATG	For insertion into pPro-Ex-HTb
STIM1_310_XhoI_rev	CGG CTCGAG CTCATTCTCAGTACCCTC	
XhoI_HAP_for	TCGAGGGAGGCTGGCGTTATTATGAA TCTTCTCTTCTTC CT TATCCTGATGGTGGT C	For insertion into GB1-TM-CC1-(His)$_6$
XhoI_HAP_rev	TCGAGACCACCATCAGGATAAGGAAGAAGAGAAGATTCAT AATAACGCCAGCCTCC C	
XhoI_LBT_for	CTCGAG GGTGGCTTTATTGAT ACCAAC	For insertion into GB1-TM-CC1-(His)$_6$
XhoI_LBT_rev	CTCGAG ACCGCCTTCTTCCAGCAGCAGTTC	

Supplementary References

1 Pham, E., Mills, E. & Truong, K. A synthetic photoactivated protein to generate local or global Ca(2+) signals. *Chem Biol* **18**, 880-890, doi:10.1016/j.chembiol.2011.04.014 (2011).

2 Senes, A., Gerstein, M. & Engelman, D. M. Statistical analysis of amino acid patterns in transmembrane helices: the GxxxG motif occurs frequently and in association with beta-branched residues at neighboring positions. *J Mol Biol* **296**, 921-936, doi:10.1006/jmbi.1999.3488 (2000).

3 Nesin, V. *et al.* Activating mutations in STIM1 and ORAI1 cause overlapping syndromes of tubular myopathy and congenital miosis. *Proceedings of the National Academy of Sciences of the United States of America* **111**, 4197-4202, doi:10.1073/pnas.1312520111 (2014).
